# Circulating tumor cells shielded with extracellular vesicle-derived CD45 evade T cell attack to enable metastasis

**DOI:** 10.1038/s41392-024-01789-1

**Published:** 2024-04-05

**Authors:** Chuan Yang, Xueping Wang, Kenneth K. W. To, Caimei Cui, Min Luo, Shaocong Wu, Lamei Huang, Kai Fu, Can Pan, Zeyu Liu, Teng Fan, Caibo Yang, Fang Wang, Liwu Fu

**Affiliations:** 1 State Key Laboratory of Oncology in South China, Guangdong Provincial Clinical Research Center for Cancer, Sun Yat-sen University Cancer Center, Guangzhou, 510060 P.R. China; 2 School of Pharmacy, Faculty of Medicine, The Chinese University of Hong Kong, Hong Kong, China; 3 LABVIV Technology (Shenzhen) Co., Ltd, Shenzhen, 518057 China

**Keywords:** Metastasis, Tumour immunology, Tumour heterogeneity

## Abstract

Circulating tumor cells (CTCs) are precursors of distant metastasis in a subset of cancer patients. A better understanding of CTCs heterogeneity and how these CTCs survive during hematogenous dissemination could lay the foundation for therapeutic prevention of cancer metastasis. It remains elusive how CTCs evade immune surveillance and elimination by immune cells. In this study, we unequivocally identified a subpopulation of CTCs shielded with extracellular vesicle (EVs)-derived CD45 (termed as CD45^+^ CTCs) that resisted T cell attack. A higher percentage of CD45^+^ CTCs was found to be closely correlated with higher incidence of metastasis and worse prognosis in cancer patients. Moreover, CD45^+^ tumor cells orchestrated an immunosuppressive milieu and CD45^+^ CTCs exhibited remarkably stronger metastatic potential than CD45^−^ CTCs in vivo. Mechanistically, CD45 expressing on tumor surfaces was shown to form intercellular CD45-CD45 homophilic interactions with CD45 on T cells, thereby preventing CD45 exclusion from TCR-pMHC synapse and leading to diminished TCR signaling transduction and suppressed immune response. Together, these results pointed to an underappreciated capability of EVs-derived CD45-dressed CTCs in immune evasion and metastasis, providing a rationale for targeting EVs-derived CD45 internalization by CTCs to prevent cancer metastasis.

## Introduction

Metastasis refers to spread of tumor from its primary site to distant organs of the body via the bloodstream or lymph vessels. It is considered the primary cause of cancer-related deaths in >90% of solid tumors.^1^ Circulating tumor cells (CTCs) are generally thought to be the metastasis precursors and they are heterogeneous at multiple levels. After CTCs sloughed off from primary tumors into the bloodstream, only a small percentage of the viable subclones could overcome shear stress, oxidative stress and escape from immune surveillance to initiate metastasis.^2^ The interaction of CTCs with immune cells in the circulation is emerging as a crucial process during metastatic progression, which has attracted a lot of attention in the cancer research community in recent years.

The mechanisms by which CTCs escape from immune surveillance are broadly classified as intrinsic and extrinsic. Intrinsically, CTCs can alter the expression of major histocompatibility complex class I (MHC I), programmed cell death ligand 1 (PD-L1), CD47, Fas/FasL to interfere with or antagonize the function of multiple immune cells.^3^ On the other hand, the extrinsic mechanisms involve CTCs homotypic clustering and the heterotypic interactions between CTCs and various blood cells.^4^ It has been reported that neutrophils interact with CTCs to form CTC-neutrophil clusters that drive cell cycle progression and escort CTCs to the metastatic sites.^5^ Besides, CTCs can also hijack platelets to avoid immune cell destruction and aid in the formation of the initial metastatic niche.^6,7^ Moreover, CTCs have also been reported to fuse with macrophages to form circulating hybrid cells overexpressing CD45, which exhibited increased tumor heterogeneity and metastatic behavior.^8^ The conventional definition of CTCs in human is a circulating cell expressing a tumor antigen (typically the epithelial cell adhesion molecule (EpCAM) or cytokeratin (CK) for epithelial cancers) but not expressing the pan-leukocyte antigen CD45.^2,9^ CD45, also known as protein tyrosine phosphatase receptor type C (PTPRC) or leucocyte common antigen (LCA), is expressed on almost all nucleated blood cells. It plays a crucial role in regulating the signaling thresholds of immune cells.^10^ Mechanistically, CD45 interacts with the antigen receptor complexes, either directly or indirectly, to regulate the T- and B-cell antigen receptor signaling. Therefore, the classical isolation approaches for CTCs would exclude the CD45-expressing macrophage-CTC hybrid cells. Recently, EpCAM^+^ CD45^+^ tumor cells have also been identified in non-small cell lung cancer (NSCLC) and serous epithelial ovarian cancer (SEOC) patients, and they were closely linked to an aggressive phenotype.^11,12^ The presence of both classical (CD45-null) CTCs and CD45-expressing macrophage-CTC hybrid cells highlights the heterogeneous tumor cells in circulation capable of seeding metastatic tumors.^8,13^

Extracellular vesicles (EVs) are membranous vesicles secreted from all living cells. They play an important role in cell-cell communication by transferring cell-derived bioactive molecules, including proteins, nucleic acids, lipids, and metabolites, and circulate in extracellular spaces.^14^ The prometastatic potential of EVs is widely recognized.^15^ For instance, exosomal PD-L1 derived from tumor cells displayed immunosuppressive effect by interacting with the PD-1 receptor on T cells.^16^ Consistently, suppression of exosomal PD-L1 was shown to reinvigorate anti-tumor immunity of T cells.^17^ Moreover, EVs derived from various immune cells were shown to elicit a variety of responses in tumor cells.^18^ Interestingly, CD45 is also found on the surface of EVs derived from immune cells.^19^ Apart from the CTC-macrophage hybrid cells with CD45 expression, CD45-positive (CD45^+^) CTCs in the circulation without hematopoietic properties but carrying malignant genotype have also been recently reported.^13^ Therefore, mechanisms other than CTC-macrophage fusion may also contribute to the formation of CD45^+^ CTCs. Importantly, CD45 plays a pivotal role in promoting CTC survival. We speculate that CTCs could uptake EVs-derived CD45 from the blood cells, thereby evading immune surveillance and disseminating in the circulation.

In this study, we set out to identify CD45^+^ CTCs in the blood samples of cancer patients and compare their metastatic potential to CD45^−^ CTCs. While evaluating the origin of CD45^+^ CTCs, we found that tumor cells could uptake EVs-derived CD45 from various immune cells, which protected them from T cell killing. In *RAB27A* knockdown or *PTPRC* genetically knockout immune cells, the secretion of EVs-derived CD45 was significantly decreased. Importantly, tumor cells preincubated with these CD45-deficient EVs were sensitive to T cell killing. Moreover, tumor cells exhibited growth advantage after intratumor injection of EVs-derived CD45 or overexpressing CD45 in immunocompetent mice but not in immunodeficient mice. Both ways were further shown to orchestrate immunosuppressive milieus within the tumor microenvironment in immunocompetent mice. Using metastatic models, CD45^+^ CTCs were shown to exhibit enhanced metastatic potential than CD45^−^ CTCs even when the number of CTCs in circulation was extremely low. Furthermore, EVs-mediated CD45 expression on tumor surfaces could trigger intercellular CD45-CD45 homophilic interactions with CD45 on T cells, subsequently preventing CD45 exclusion from TCR-pMHC synapse. T cell receptor (TCR) signaling was therefore inhibited to suppress immune surveillance. These results suggested potential implications for targeting EVs-derived CD45 internalization to prevent cancer metastasis.

## Results

### CD45^+^ CTCs were unequivocally identified in blood sample from cancer patients

**Fig. 1:**
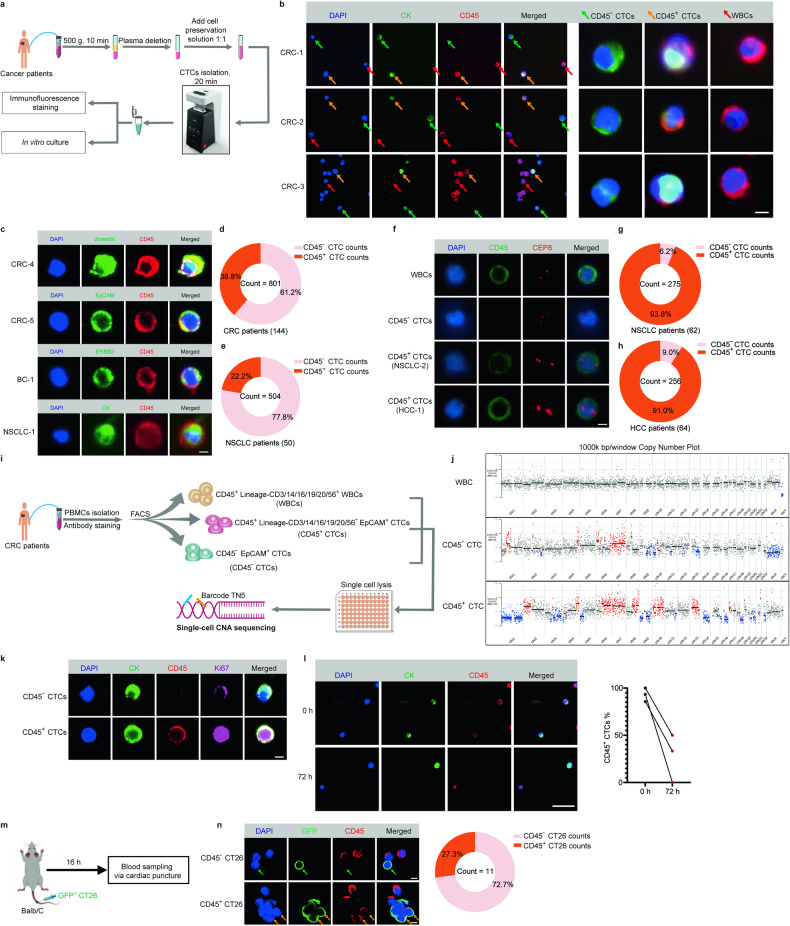
CD45^+^ CTCs were unequivocally identified in blood samples from cancer patients. **a** Schematic diagram showing the isolation of CTCs from blood samples of cancer patients using the Cellab Thomas I CTCs processing workstation. **b** Representative images of CD45^−^ CTCs and CD45^+^ CTCs isolated from blood samples of CRC patients. CRC-1, CRC-2 and CRC-3 are three randomly selected CRC patients. Isolated CTCs were stained with pan-cytokeratin (CK, green), CD45 (red) and DAPI (nuclei, blue). Green arrows represent CD45^−^ CTCs, Golden arrows represent CD45^+^ CTCs, and red arrows represent WBCs. Scale bar = 5 μm. **c** Representative images of CD45^+^ CTCs isolated from two CRC patients (CRC-4 and CRC-5) stained with Vimentin (green) and EpCAM (green), a breast cancer patient (BC-1) stained with ERBB2 amplification (green); and a NSCLC patient (NSCLC-1) stained with CK (green), all of which were stained with CD45 (red) and DAPI (nuclei, blue). Scale bar = 5 μm. **d**, **e** Pie chart summarizing the number of CTC counts among 144 CRC patients and 50 NSCLC patients using the Cellab Thomas I CTCs processing workstation. **f** Representative images of WBCs, CD45^−^ CTCs and CD45^+^ CTCs using CEP8 staining (Red), CD45 (Green), DAPI (Blue). Scale bar = 5 μm. **g**, **h** Pie chart summarizing the number of CTC counts among 62 NSCLC patients and 64 HCC patients using NextCTC capturing CTCs. **i** Procedures of single-cell CNA sequencing for sorted cells. **j** Representative copy number plots (1000k bp/window) of three-type cells after single-cell CNA sequencing. **k** Representative images of Ki67 staining for CD45^−^ CTCs and CD45^+^ CTCs after 72 h in vitro culture. Scale bar = 5 μm. **l** The change in the percentage of CD45^+^ CTCs (100% × CD45^+^ CTCs count/ total CTCs count) in 3 CRC patients before (0 h) and after in vitro culture (72 h). Scale bar = 100 μm. **m** Schematic diagram showing the procedure for mouse CTCs acquisition after injection of GFP^+^ CT26 cells in the mouse tail vein. **n** Representative images of CD45^−^ GFP^+^ CT26 cells (Green arrow) and CD45^+^ GFP^+^ CT26 cells (Golden arrow) from mouse blood sample via cardiac puncture, CD45 (red) and DAPI (blue). Scale bar = 10 μm

CTCs were traditionally defined as EpCAM-isolated intact cells showing positive staining for CK but negative staining for CD45.^20^ This conventional definition has inevitably ignored CD45 positive cells with cancer cell characteristics.^2,9^ To determine whether CD45^+^ CTCs exist in the circulation of cancer patients, we first isolated CTCs from the peripheral blood of colorectal cancer (CRC) patients using Cellab Thomas I CTCs processing workstation (Fig. 1a). Both CD45^+^ CTCs and CD45^−^ CTCs were detected simultaneously by immunofluorescence staining with CD45 and the epithelial marker pan-cytokeratin (CK) (CD45^+^ CTCs were CK positive and CD45 positive whereas CD45^−^ CTCs were CK positive but CD45 negative) (Fig. 1b). Moreover, we investigated the presence of CD45^+^ CTCs in CRC, breast cancer (BC) and NSCLC patients by detecting the epithelial-mesenchymal markers EpCAM, Vimentin, or abnormal expression of ERBB2, or CK (Fig. 1c). Blood samples from a cohort of 144 CRC patients who were diagnosed for the first time or who developed progressive disease even after extensive treatment were obtained (Supplementary Table 1). From the blood samples (5 mL) of the 144 CRC patients, a total of 801 CTCs were detected including 311 CD45^+^ CTCs (38.8%) and 490 CD45^−^ CTCs (61.2%) (Fig. 1d). On the other hand, in another cohort of 50 NSCLC patients using Cellab Thomas I CTCs processing workstation, 112 CD45^+^ CTCs (22.2%) and 392 CD45^−^ CTCs (77.8%) were detected (Fig. 1e, Supplementary Table 2). To further unbiasedly verify the existence of CD45^+^ CTCs, chromosome centromere probe 8 (CEP8), another important marker of CTCs was independently used to evaluate these CTCs by using NextCTC capturing CTCs. Likewise, CEP8^+^ CD45^+^ CTCs (CEP8 positive was defined as red phosphor dots ≥ 3 in the cell nucleus) were detected in NSCLC and hepatocellular carcinoma (HCC) patients (Fig. 1f–h).

Copy number alteration (CNA) is an important characteristic of cancer. To determine whether these CD45^+^ cells originated from cancer, we sorted three populations of single cells from three colorectal cancer patients by FACS: (a) CD45^+^ Lineage-APC^+^ WBCs (Lineage contains CD3/14/16/19/20/56); (b) CD45^+^ Lineage-APC^-^ EpCAM^+^ CTCs; (c) CD45^−^ EpCAM^+^ CTCs. 65 cells (including 23 WBCs, 24 CD45^+^ CTCs and 18 CD45^−^ CTCs) were performed single-cell CNA sequencing (Supplementary Fig. 1a, Fig. 1i). After incorporating qualified sample by Coefficient of Variation (CV < 0.5) and Median Bin Count (MBC > 35), 23 WBCs, 6 CD45^+^ CTCs and 7 CD45^−^ CTCs were used for further analysis. All 23 WBCs had consistent low CNA scores, however, CD45^+^ CTCs and CD45^−^ CTCs showed significant high CNA scores, indicating the cancer origin CD45^+^ CTCs (Fig. 1j).

Since CD45 is specifically expressed in blood cells but not tumor cells, we speculated that CD45 was not produced by CD45^+^ CTCs themselves. Hence, we sorted CTCs and calculated the change of CD45^+^ CTCs percentage before (0 h) and after in vitro culture (72 h) of the sorted cells. The proliferative activity of these CTCs was detected and the results showed both CD45^−^ CTCs and CD45^+^ CTCs kept proliferative state after 72 h in vitro culture (Fig. 1k). However, in CTC samples sorted from 3 randomly selected CRC patients, a remarkable decrease in the percentage of CD45^+^ CTCs was observed (Fig. 1l). In addition, we performed tail vein injection of CD45^−^ GFP^+^ CT26 cells into the circulation of Balb/C mice (Fig. 1m). After 16 h, blood sample was collected from the mice via cardiac puncture. Interestingly, the coexistence of CD45^−^ GFP^+^ CT26 cells and CD45^+^ GFP^+^ CT26 cells was observed, with the latter accounting for 27.3% of the total GFP^+^ CT26 cell population (Fig. 1n).

These results suggested that the CD45^+^ CTCs was a previously overlooked subtype of CTCs that could be converted from CD45^−^ CTCs.

### CD45^+^ CTCs were endowed with significantly higher metastatic potential

**Fig. 2:**
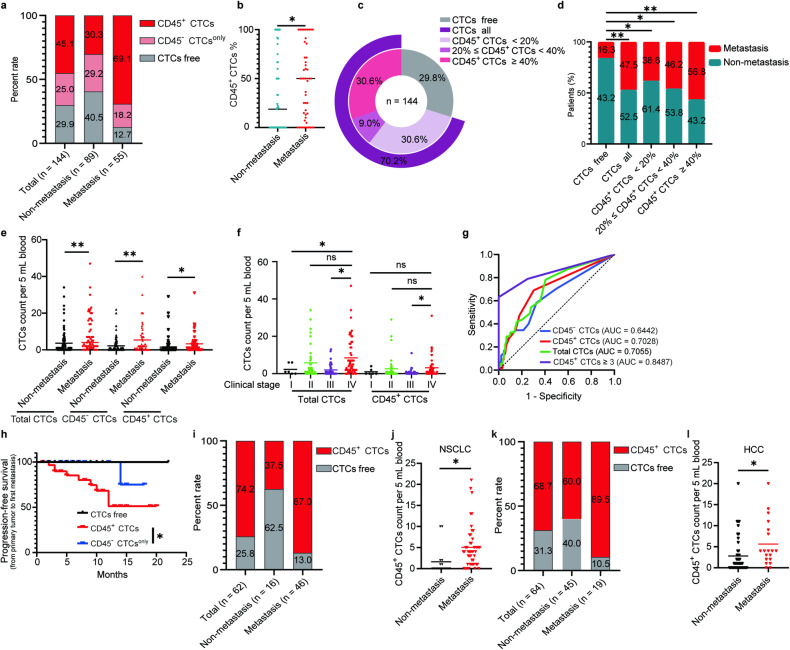
Presence of CD45^+^ CTCs is closely associated with high metastatic potential. **a** Bar chart summarizing CD45^+^ CTCs (CD45^+^ CTC counts ≥ 1 cell per 5 mL blood) and CD45^−^ CTCs^only^ (CD45^−^ CTC counts ≥ 1 cell without CD45^+^ CTCs per 5 mL blood) statistics among 144 CRC patients with or without metastasis. The definition of CD45^+^ CTCs and CD45^−^ CTCs^only^ is similar hereafter. **b** The percentage of CD45^+^ CTCs (CD45^+^ CTCs % = 100% × CD45^+^ CTCs / total CTCs) among 144 CRC patients with or without metastasis. **c** Pie chart showing the composition of 144 CRC patients according to the percentage of CD45^+^ CTCs. **d** Metastasis rates among 144 CRC patients with different proportions of CD45^+^ CTCs. *P < 0.05, **P < 0.01 by Chi-square (and Fisher’s exact) test. **e** CTC counts of CD45^+^ CTCs and CD45^−^ CTCs among 144 CRC patients with or without metastasis. **f** CTC counts among CRC patients in different clinical stages. **g** Receiver operating characteristic (ROC) curve showing the sensitivity and specificity of CD45^+^ CTCs versus CD45^−^ CTCs or Total CTCs, CD45^+^ CTCs ≥ 3 for predicting metastasis. “CD45^+^ CTCs ≥ 3” means CD45^+^ CTC count is equal to or more than 3 per 5 mL blood sample of one patient. In the group of CD45^+^ CTCs ≥ 3, patients were defined as “non-metastasis” when they were metastasis-free for at least one-year during the follow-up. **h** Kaplan-Meier plot showing progression-free survival of patients with CD45^+^ CTCs or CD45^−^ CTCs. *P* value by two^-^sided log-rank test is 0.0364. **i**–**l** Two cohorts of CD45^+^ CTCs from 62 NSCLC and 64 HCC cancer patients using CEP8 staining and counting with NextCTC capturing CTCs. **i** Bar chart summariz**i**ng CD45^+^ CTCs statistics among 62 NSCLC patients with or without metastasis. **j** CTC counts of CD45^+^ CTCs among 62 NSCLC patients with or without metastasis. **k** Bar chart summarizing CD45^+^ CTCs statistics among 64 HCC patients with or without metastasis. **l** CTC counts of CD45^+^ CTCs among 64 HCC patients with or without metastasis. All dot graph data presented as *P < 0.05, **P < 0.01 were acquired by nonparametric Mann-Whitney *U* test

While CTCs are known to drive tumor metastasis, they are highly heterogeneous. We investigated whether the CD45^+^ CTCs have a higher tendency of spreading. Among the 144 CRC patients, 101 (70.1%) patients had detectable CTCs including 36 patients (25.0%) with CD45^−^ CTCs (CD45^−^ CTCs^only^) and 65 patients (45.1%) with CD45^+^ CTCs, with a mean number of 8 CTCs per 5 mL of blood sample. A further sub-group analysis revealed that among 89 patients without metastasis, 53 (59.5%) patients had detectable CTCs, with 26 patients (29.2%) having CD45^−^ CTCs^only^ and 27 patients (30.3%) having CD45^+^ CTCs. On the other hand, in the other 55 patients with metastasis, 10 patients (18.2%) had CD45^−^ CTCs^only^ and 38 patients (69.1%) had CD45^+^ CTCs (Fig. 2a). Therefore, CD45^+^ CTCs were more prevalent in metastatic patients (69.1%) than in early-stage cancer patients without metastasis (30.3%) among the 144 CRC patients. Intriguingly, patients with metastasis had a significantly higher percentage of CD45^+^ CTCs (CD45^+^ CTCs % = 100% × CD45^+^ CTC counts/total CTC counts) than those without metastasis (Fig. 2b).

Next, we sought to ascertain the correlation between high CD45^+^ CTCs percentage (CD45^+^ CTCs^hi^ defined as CD45^+^ CTC counts / total CTC counts ≥ 40% per 5 mL blood sample from each cancer patient) and metastasis. It is noteworthy that CRC patients with CTCs (CTCs all) had a significantly higher metastasis rate (47.5%) than those without CTCs (CTCs free, 16.3%). More importantly, CD45^+^ CTCs^hi^ group (44 patients, 30.6%) was found to make up 56.8% of the metastatic patients. In contrast, the CD45^+^ CTCs^low^ group (CD45^+^ CTCs^low^ defined as CD45^+^ CTC counts / total CTC counts < 20% cells per 5 mL blood sample from each cancer patient, 44 patients, 30.6%) exhibited a metastatic rate of only 38.6% (Fig. 2c, d).

High CTC counts were strongly associated with poor prognosis in several cancer types.^2,21,22^ The correlation between CTC count and metastasis was investigated in our CRC patient cohort. Patients with advanced metastatic cancer were shown to have significantly higher total CTCs, CD45^+^ CTCs and CD45^−^ CTCs than those with non-metastatic cancer (Fig. 2e). Furthermore, late-stage CRC patients had significantly higher CTC counts (including both CD45^+^ CTCs and CD45^−^ CTCs) than early-stage cancer patients (Fig. 2f).

We then investigated whether the presence of CD45^+^ CTCs in blood samples of CRC patients could predict metastasis and prognosis. A receiver-operating characteristic (ROC) analysis was performed to evaluate the predictive ability of total CTCs, CD45^+^ CTCs and CD45^−^ CTCs for metastasis. The ROC curve (a plot of sensitivity versus 1-specificity of a diagnostic test) evaluating the predictive ability of the CTC subgroups for metastasis was shown in Fig. 2g. Remarkably, CD45^+^ CTCs had higher accuracy and superiority in predicting metastasis, with an area under the curve (AUC) of 0.7028 (*p* < 0.0001) compared to CD45^−^ CTCs having an AUC of 0.6442 (*P* = 0.0037) (Fig. 2g). When simultaneously calculating CD45^−^ CTCs and CD45^+^ CTCs (Total CTCs), the AUC was 0.7055 (*P* < 0.0001) (Fig. 2g). Next, we investigated whether a higher CD45^+^ CTC counts was associated with a stronger prognostic potential for metastasis. When the CD45^+^ CTC counts was equal to or more than 3 per 5 mL blood sample from individual patient, the AUC in the ROC curve reached its maximum value of 0.8487 (*P* = 0.0316) (Fig. 2g). Furthermore, the progression free survival (PFS) of patients bearing CD45^+^ CTCs or CD45^−^ CTCs^only^ or CTC free was also evaluated. The CD45^+^ CTC patient group was shown to exhibit significantly worse PFS than the CD45^−^ CTC patient group (Fig. 2h).

Moreover, using CEP8 staining and counting, CD45^+^ CTCs were verified correlating with metastasis in two independently cohorts of 62 NSCLC patients and 64 HCC patients (Fig. 2i–l, Supplementary Table 3, Supplementary Table 4).

Taken together, the presence of CD45^+^ CTCs were demonstrated in patients bearing various cancer types (CRC, NSCLC and HCC) and they were significantly associated with more extensive metastasis and poor prognosis.

### Tumor cells could internalize WBC-derived EVs containing CD45

**Fig. 3:**
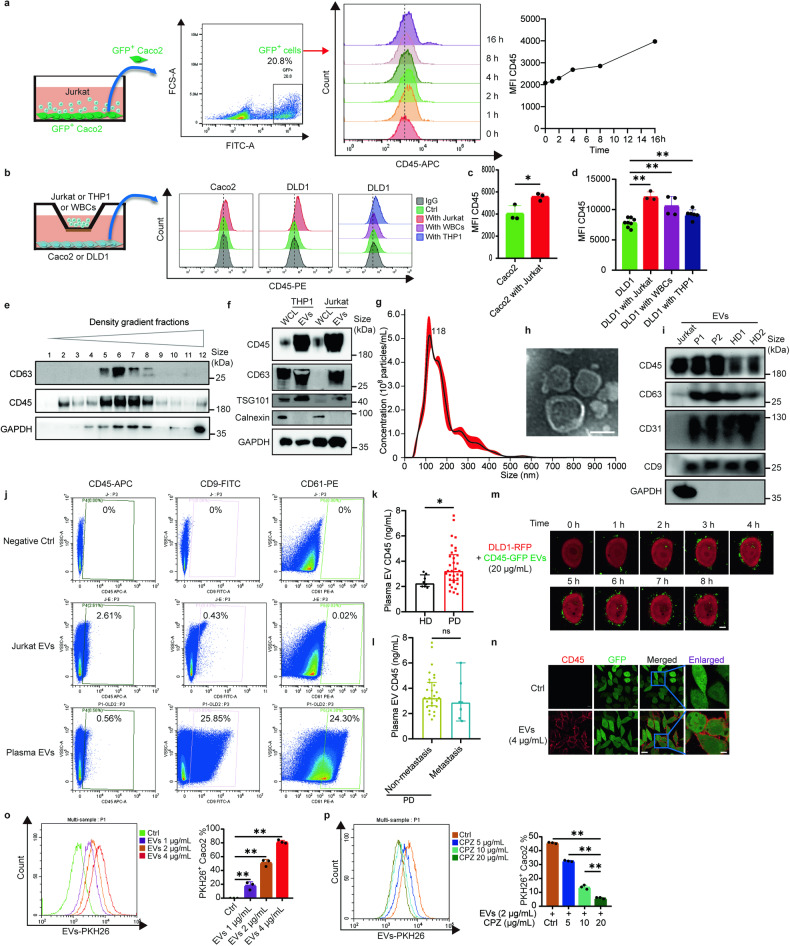
CD45 shed from WBCs could be transferred via EVs to tumor cells. **a** GFP^+^ Caco2 cells co-cultured with Jurkat cells at a ratio of 1:4 for different time (0, 1, 2, 4, 8, 16 h), then GFP^+^ Caco2 cells were collected and stained with anti-CD45-APC to dynamically analyze the MFI shifting of CD45 among GFP^+^ Caco2 cells using flow cytometry. **b** Flow cytometric analysis of CD45 transfer from Jurkat cells to Caco2 cells, or from Jurkat cells/ THP1 cells/ WBCs to DLD1 cells following 16 h of indirect co-culture. **c**, **d** Bar graph showing the MFI of CD45-PE of Caco2 cells or DLD1 cells after 16 h of indirect co-culture with Jurkat cells/ THP1 cells/ WBCs. **e**, **f** Immunoblot analysis showing the makers of EVs isolated from supernatant of THP1 and Jurkat cells. **e** Density gradient fractions of EVs from Jurkat cells. **f** Positive (TSG101 and CD63) and negative (Calnexin) markers of EVs. WCL: whole cell lysis. Each ladder was loaded with protein 30 μg. **g** Nanoparticle tracking analysis of Jurkat cell-derived EVs. **h** Transmission electron microscope imaging of Jurkat cell-derived EVs. Scale bar = 50 nm. **i** Plasma EVs charactering by immunoblotting. Jurkat-EVs was used as positive control. P1- and P2-EVs were examples of two CRC cancer patients, HD1 and HD2 were examples of two healthy donors. **j** Plasma EVs charactering by nano-flow cytometry analysis. **k, l** CD45 ELISA assay for the measurement of EVs-derived CD45 from healthy donors (HD) and CRC patients (PD) with or without metastasis. **m** Time series of live cell imaging of DLD1-RFP cells after the addition of EVs (20 μg/mL) derived from CD45-GFP-expressing HEK293T cells for up to 8 h. Scale bar = 5 μm. **n** Immunofluorescence images of GFP^+^ Caco2 cells after incubation with PBS or EVs isolated from Jurkat cell supernatant for 12 h. Left scale bar = 20 μm, right scale bar = 5 μm. **o** Flow cytometric analysis for the measurement of Jurkat cell-derived EVs uptake by Caco2 cells. Caco2 cells were incubated with different concentration of PKH26-stained EVs (0, 1, 2, or 4 μg/mL) for 12 h before analysis. **p** Caco2 cells were incubated with PKH26-stained EVs (2 μg/mL) and chlorpromazine (CPZ, 0, 5, 10, or 20 μg/mL) for 12 h before collection for flow cytometric analysis of PKH26^+^ Caco2 cells. All bar graph data are presented as means ± SEM. *P < 0.05, **P < 0.01 by Student’s *t* test or Mann-Whitney *U* test

CD45 is a unique and ubiquitous membrane glycoprotein expressed on almost all nucleated hematopoietic cells but is not present on epithelial cells. Tumor cells were reported to acquire immune regulatory molecules from tumor-infiltrating lymphocytes via trogocytosis or exosome transfer.^23,24^ Moreover, in the circulating CT26 tumor study above, CD45 expression was detected on the surface of CD45^−^ CT26 cells after tail vein injection of the tumor cells into the circulation of Balb/C mice (Fig. 1g). Therefore, we speculated that the CD45 on the surface of CD45^+^ CTCs was derived from white blood cells (WBCs) rather than being expressed endogenously. To test this hypothesis, we conducted direct and indirect co-culture system containing tumor cells and blood cells, respectively. In the direct co-culture system, GFP-overexpressing Caco2 (GFP^+^ Caco2) cells were allowed to co-culture directly with Jurkat cells suspending on the medium (Fig. 3a). CD45 expression was found to be enriched progressively in GFP^+^ Caco2 cells in co-culture with Jurkat cells for up to 16 h (Fig. 3a). In the indirect co-culture system, adherent Caco2 or DLD1 cancer cells were allowed to co-culture with Jurkat, THP1, or WBCs placed in permeable support cell culture inserts (Fig. 3b). As detected by flow cytometry, DLD1 and Caco2 cells exhibited significant higher CD45 enrichment on their surface when they were indirectly co-cultured indirectly with WBCs, Jurkat, or THP1 cells for 16 h (Fig. 3b–d). The data suggested that tumor cells could acquire CD45 in a paracrine manner, presumably mediated by EVs. To this end, CD45 was shown to be enriched in EVs collected from Jurkat cell-conditioned medium using sucrose density gradient centrifugation and immunoblotting (Fig. 3e). The identity and purity of the EVs derived from THP1 and Jurkat cells were confirmed by immunoblotting with the EVs-derived markers (CD63 and TSG101) (Fig. 3f). Calnexin was also detected as a negative marker for EVs. In addition, Jurkat cell-derived EVs were further confirmed by nanoparticle tracking analysis and transmission electron microscope imaging (Fig. 3g, h). Furthermore, the presence of CD45 in EVs collected from plasma samples of healthy donors or cancer patients was also confirmed by immunoblotting, nano-flow cytometry and ELISA assay (Fig. 3i–l). Cancer patients were found to express higher level of CD45 on their plasma EVs than healthy donors (Fig. 3k). On the other hand, CD45 level in the plasma from non-metastatic and metastatic cancer patients was not significantly different (Fig. 3l).

To determine whether EVs-derived CD45 could be transferred to tumor cells, cancer cells were incubated with EVs and the internalization of EVs into cancer cells was detected by immunofluorescence and flow cytometry. To visualize the internalization of EVs-derived CD45, live cell imaging of DLD1-RFP cells was conducted over 8 h when they were incubated with EVs from CD45-GFP fusion protein-expressing HEK293T cells. The uptake of EVs-derived CD45 was detected as the GFP fluorescent puncta entered the DLD1-RFP cells in a time-dependent manner (Fig. 3m). The maximum EVs-derived CD45-GFP uptake was observed in about 4–6 h and, thereafter EVs-derived CD45 level was maintained stable for at least 4 h (Fig. 3m). Similarly, cellular uptake of EVs-derived CD45 was also observed in GFP^+^ Caco2 cells (having negligible CD45 expression) after incubation with Jurkat-derived EVs for 12 h (Fig. 3n). By flow cytometric detection, the cellular internalization of PKH26-stained EVs into Caco2 cells was shown to be concentration-dependent (Fig. 3o). In addition, to exclude the expression of CD45 endogenously, qRT-PCR was performed to measure the transcription levels of CD45. Jurkat cells had high transcription level of CD45 (mean ct value = 23.8), while tumor cells including Caco2, DLD1 and THC8307 cells had low expression of CD45 at the transcription level (ct value > 35). Besides, we didn’t observe significant transcription differences of CD45 after they were incubated with EVs for 16 h. This indicates CD45 is not endogenously produced by tumor cells (Supplementary Fig. 2a). Moreover, EVs uptake was significantly decreased by chlorpromazine (CPZ, a pharmacological inhibitor of Clathrin critical for EVs uptake) (Fig. 3p), thus further confirming the tumor cell uptake of CD45 via EVs. Importantly, CTCs in the circulation suffer from anoikis, shear stress, oxidative stress and immune surveillance. We mimicked these circumstances to see which condition would force tumor cells to absorb more EVs containing CD45. These methods including adding H_2_O_2_ to create oxidative stress, adding IFN-γ to increase immune pressure, centrifugating to give rise to anoikis and shear stress. Interestingly, centrifugation contributed the most significant more absorption value of EVs-CD45 (Supplementary Fig. 2b). Therefore, it’s possible that CTCs in the circulation can absorb enough EVs-CD45.

### CD45-shielded tumor cells were resistant to T cell killing

**Fig. 4:**
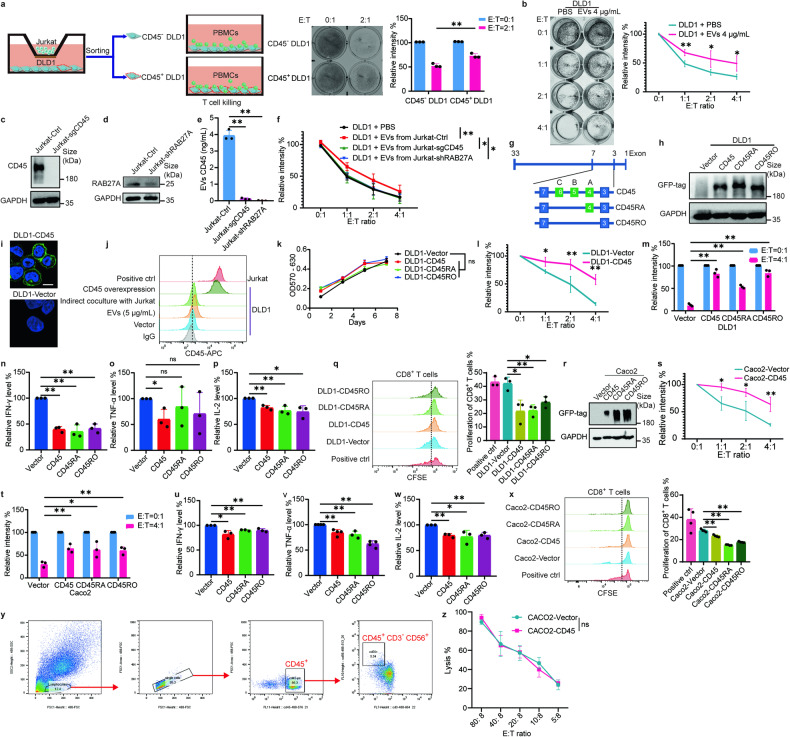
CD45 overexpressing tumor cells were resistant to T cell killing. **a** T cell killing effect was tested in DLD1 cells that had been indirectly pre-cocultured with Jurkat cells. For the evaluation of T cell killing, PBMCs (effector; E) were incubated with CD45^−^ or CD45^+^ DLD1 cells (tumor cell; T) at a E:T ratio of 2:1. **b** T cell cytotoxicity assay of DLD1 cells (preincubated with PBS or Jurkat cell-derived EVs) in co-culture with PBMCs after the addition of OKT3 (1 μg/mL) at various E:T ratios. The images on the left are representative images of survived DLD1 cells stained with crystal violet, and the graph on the right is a line graph showing the relative intensity of survived DLD1 cells. **c**, **d** Immunoblot analysis showing CD45 expression after CRISPR Cas9 knockout of *PTPRC* or *RAB27A* knockdown in Jurkat cells. **e** Bar graph showing EVs-derived CD45 levels from supernatant of Jurkat cells by ELISA assay. **f** T cell cytotoxicity assay of DLD1 cells (preincubated with PBS or Jurkat cell-derived EVs (EVs from Jurkat-Ctrl, Jurkat-sgCD45 or Jurkat-shRAB27A cells) in co-culture with PBMCs after the addition of OKT3 (1 μg/mL) at different E:T ratios. **g** Schematic diagram showing the different CD45 isoforms tested in the study. **h** Immunoblot analysis of DLD1 cells overexpressing different CD45 isoforms. **i** Immunofluorescence imaging of membrane CD45-GFP on DLD1 cells. Scale bar = 5 μm. **j** Comparison of CD45 expression on DLD1 cells and Jurkat cells. **k** Proliferation assay of DLD1 cells overexpressing different isoforms of CD45. **l, m** T cell cytotoxicity assay of DLD1 cells with or without CD45 overexpression co-cultured with PBMCs in the presence of pre-coated OKT3 (1 μg/mL) at different E:T ratios. **n**–**p** Quantitative analysis of IFN-γ, TNF-α, and IL-2 secretion in the co-culture system of T cell cytotoxicity assay with DLD1 cells. **q** Proliferation of carboxyfluorescein succinimidyl ester (CFSE)-labeled CD8^+^ T cells in the co-culture system of T cell cytotoxicity assay with DLD1 cells. **r** Immunoblot analysis of Caco2 cells overexpressing different CD45 isoforms. **s, t** T cell cytotoxicity assay of Caco2 cells with or without CD45 overexpression co-cultured with PBMCs in the presence of pre-coated OKT3 (1 μg/mL) at different ratios. **u**–**w** Quantitative analysis of IFN-γ, TNF-α, and IL-2 secretion in the co-culture system of T cell cytotoxicity assay with Caco2 cells. **x** Proliferation of CFSE-labeled CD8^+^ T cells in the co-culture system of T cell cytotoxicity assay with Caco2 cells. **y** NK cell sorting strategy from WBCs. **z** Lysis percentage of NK cell killing Caco2 cells with density diluted NK cells at NK: Tumor ratios of 80: 8 to 5: 8. All bar graph data are presented as means ± SEM. *P < 0.05, **P < 0.01 by Student’s *t* test or Mann-Whitney *U* test

CD45 is a well-established regulator of immune cell signaling thresholds.^10^ The expression of CD45 on tumor cell surface may also play an important immunomodulatory role. To determine whether the acquisition of CD45 on CTCs contributed to immune evasion, the T cell killing effect was examined by incubating PBMCs from healthy donors with CD45^+^ DLD1 cells sorted from parent DLD1 cells after previous indirect coculture with Jurkat cells (Fig. 4a). CD45^+^ DLD1 cells were found to be more resistant to T cell killing compared to CD45^−^ DLD1 cells (Fig. 4a). Similarly, DLD1 cells preincubated with EVs from Jurkat cells (thus acquiring CD45), were also found to be more resistant to T cell killing than DLD1 cells without EVs preincubation (Fig. 4b). To ascertain the role of EVs-derived CD45 in the observed T cell killing effect, we generated *PTPRC*-deficient Jurkat cells (Jurkat-sgCD45) lacking CD45 expression by CRISPR Cas9 technology and *RAB27A*-knockdown Jurkat cells (Jurkat-shRAB27A) that were deficient in EVs secretion (Fig. 4c–e). Unlike the CD45^+^ DLD1 cells, DLD1 cells preincubated with EVs from Jurkat-sgCD45 or Jurkat-shRAB27A cells were found to be sensitive to CD8^+^ T cell-mediated T cell killing (Fig. 4f). These results indicated that EVs-mediated CD45 expression on the surface of tumor cells could shield CTCs from immune surveillance and suppress the T cell killing response.

To investigate how tumor cells utilize CD45 molecules to escape from T cell killing, we transfected DLD1 cells with different isoforms of CD45-GFP fusion constructs, including the longest isoform (CD45RABC, CD45 in short), the resting isoform expressing on naive T cells (CD45RA) and the activated isoform specific to effector and memory T cells (CD45RO) (Fig. 4g–k). DLD1 cells ectopically overexpressing the above CD45 isoforms were consistently more resistant to T cell killing than the control group (Fig. 4l, m). However, there was no appreciable differences in cell proliferation rate of DLD1 cells overexpressing the 3 different CD45 isoforms (Fig. 4k). Moreover, a significant reduction in cytokine secretion (IFN-γ, TNF-α, IL-2) was noted in supernatants from co-culture system of CD8^+^ T cells and DLD1 cells overexpressing each of the three CD45 isoforms, indicating attenuated T cells activation (Fig. 4n–p). Though we observed a few differences about TNF-α secretion in the CD45RA and CD45RO groups compared to control group (Fig. 4o). Furthermore, the proliferation potential of carboxyfluorescein diacetate succinimidyl ester (CFSE)-labeled CD8^+^ T cells was decreased when they were co-cultured with DLD1 cells overexpressing CD45 (Fig. 4q). In another cancer cell line Caco2, a similar resistance phenotype to T cell killing was also observed when Caco2 cells overexpressed the CD45 isoforms (Fig. 4r–x).

NK-cells also play perplexing roles in the immune destruction of CTCs. To verify whether tumor cells overexpressing CD45 are more resistant to NK-cell mediated lysis, NK-cells were isolated from peripheral blood of healthy donors by FACS with the population of CD45^+^ CD3^-^ CD56^+^ cells (Fig. 4y). Density diluted NK cells were co-cultured with Caco2 cells in the presence of IL-2 and IL-15 for 4 h. We didn’t observe significant differences of tumor cell lysis between Caco2-Vector and Caco2-CD45 cells (Fig. 4z). This suggested that the mechanisms of CD45^+^ CTCs evading immune surveillance may not be dependent on resisting NK-cell mediated killing.

Collectively, our data suggested that EVs-mediated acquisition of CD45 expression on cancer cells could lead to immune evasion by attenuating T cell killing.

### CD45-overexpressing tumor cells coordinated an immunosuppressive milieu in vivo

**Fig. 5:**
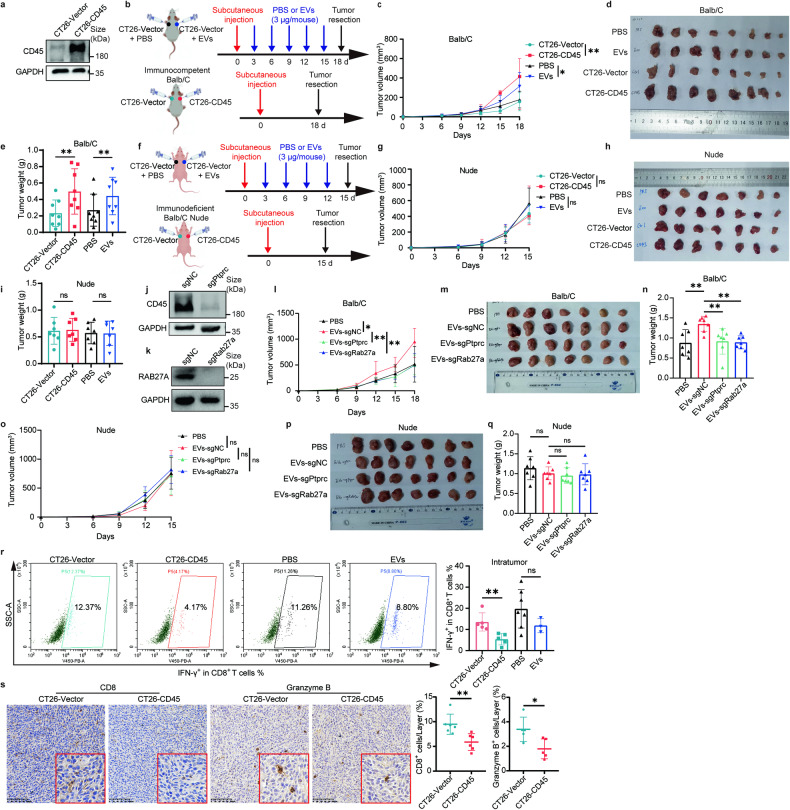
Tumor cells acquiring CD45 orchestrated an immunosuppressive milieu in vivo. **a** Immunoblot analysis confirming CD45 overexpression in CT26-CD45 cells. **b** Schematic diagram showing the treatment procedure of the subcutaneous tumor model in immunocompetent Balb/C mice. **c** Tumor growth curves in immunocompetent Balb/C mice on the indicated days with CT26-Vector or CT26-CD45 cells (*n* = 8). **d, e** Image showing tumor size of immunocompetent Balb/C mice and bar graph showing tumor weight at the endpoint of the experiment (*n* = 8). **f** Schematic diagram showing the treatment procedure of subcutaneous tumor model in immunodeficient Balb/C nude mice. **g** Tumor growth curves in the immunodeficient Balb/C nude mice on the indicated days with CT26-Vector or CT26-CD45 cells (*n* = 7). **h, i** Image showing tumor size of immunodeficient Balb/C nude mice and bar graph showing tumor weight at the endpoint of the experiment (*n* = 7). **j, k** Immunoblotting analysis of the knockout efficiency of CD45 and Rab27a in Raw 264.7 cells, separately. **l** Tumor growth curves of CT26 cells in immunocompetent Balb/C mice on the indicated days with intratumor injection of Raw 264.7-derived EVs (EVs-sgNC), Raw 264.7-sgPtprc-derived EVs (EVs-sgPtprc), as well as Raw 264.7-sgRab27a-derived EVs (EVs-sgRab27a) every 3 days (*n* = 7). **m, n** Image showing tumor size of immunocompetent Balb/C mice and bar graph showing tumor weight at the endpoint of the experiment (*n* = 7). **o**–**q** Repeated experiments similar to (**l**–**n**) in immunodeficient Balb/C nude mice (*n* = 7). **r** Flow cytometric analysis of IFN-γ^+^ CD8^+^ T cells from tumor tissues of immunocompetent Balb/C mice. **s** Representative image of immunohistochemistry staining of infiltrated CD8 positive and granzyme B positive cells in tumor tissue (Left) and relative quantification of infiltrated CD8 positive and granzyme B positive cells in tumor tissue (Right). Each layer contained 3 to 8 field of view. Scale bar = 100 μm. Images in red frame were 2× magnified. All bar graph data are presented as means ± SEM. *P < 0.05, **P < 0.01 by Student’s *t* test or Mann-Whitney *U* test

To further evaluate the proliferation potential and immunomodulatory role of CD45-overexpressing tumor cells in vivo, we transfected CT26 cells with vectors encoding mouse CD45 proteins and compared the tumorigenic ability of CT26-Vector cells and CT26-CD45 cells in immunocompetent Balb/C mice or immunodeficient Balb/C nude mice (Fig. 5a). In immunocompetent Balb/C mice, CT26-CD45 cells were found to grow significantly faster than CT26-Vector cells when they were injected subcutaneously to the opposite flank of the same mouse, respectively (Fig. 5b–e). However, when CT26-Vector cells and CT26-CD45 cells were subcutaneously injected in immunodeficient Balb/C nude mice, both of them showed similar growth potential (Fig. 5f–i). The finding indicated that CT26-CD45 cells acquired growth advantage over CT26-Vector cells in immunocompetent mice. Besides, subcutaneously administered Raw 264.7-derived EVs containing CD45 promoted growth of CT26 cells in immunocompetent Balb/C mice but not immunodeficient Balb/C nude mice (Fig. 5b–i). Moreover, CD45-knockout and Rab27a-knockout Raw 264.7 cells were used to collect their EVs for intratumor injection. The effect of tumor promoting from EVs-sgNC in immunocompetent Balb/C mice was not seen from EVs-sgPtprc, neither EVs-sgRab27a (Fig. 5j–q, Supplementary Fig. 3a). Furthermore, in immunocompetent Balb/C mice, there was a significant reduction in IFN-γ^+^ CD8^+^ T cells in the tumor xenograft developed from CT26-CD45 than CT26-Vector cells, implying an immunosuppressive environment (Fig. 5r). In addition, by immunohistochemistry staining, there was also remarkably less infiltration of CD8 positive and granzyme B positive immune cells in the tumor tissues developed from CT26-CD45 than CT26-Vector cells (Fig. 5s). Taken together, the findings suggested CD45 expression on tumor cells played an immunosuppressive role and allowed tumors to evade immune surveillance.

### CD45^+^ CTCs represented the metastatic precursors in vivo

**Fig. 6:**
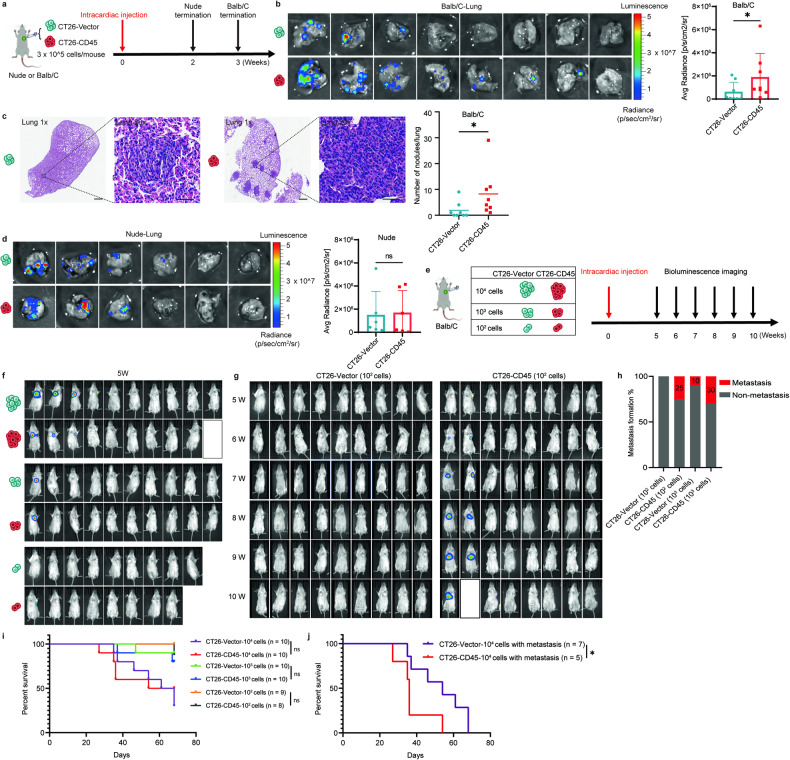
CD45^+^ CTCs represent the metastatic precursors in vivo. **a** Schematic diagram depicting establishment of in vivo metastasis model by intracardiac injection of CT26-Vector or CT26-CD45 cells in immunocompetent Balb/C mice. **b** Live imaging for visualization and quantification of the lung metastasis from Balb/C mice (*n* = 8). **c** Representative H&E staining images from lung tissue of Balb/C mice (left). Scale bar = 0.5 mm or 50 μm. The bar graph on the right summarizes the number of metastatic nodules in the lung of Balb/C mice (*n* = 8). **d** Live imaging for visualization and quantification of the lung metastasis from Balb/C nude mice (*n* = 6). **e** Schematic diagram depicting the procedure for the in vivo metastatic model established by intracardiac injection of CT26-Vector or CT26-CD45 cells to immunocompetent Balb/C mice using limiting dilution method. **f** Live imaging of immunocompetent Balb/C mice after intracardiac injection of CT26-Vector or CT26-CD45 cells (10^2^, 10^3^, or 10^4^ cells per mouse) at 5 weeks. **g** Live imaging of mice after intracardiac injection of 10^2^ CT26-Vector cells or 10^2^ CT26-CD45 cells from 5 to 10 weeks. **h** Bar graph showing metastatic rate of mice at the endpoint of the experiment. CT26-Vector (10^2^ cells) group is 9 mice, CT26-CD45 (10^2^ cells) group is 8 mice, and the numbers of mice in CT26-Vector (10^3^ cells) and CT26-CD45 (10^3^ cells) group were both 10. **i** Kaplan-Meier plot showing the overall survival of mice injected with different limiting dilution of CT26-Vector or CT26-CD45 cells. **j** Kaplan-Meier plot showing the overall survival of mice with metastasis in the endpoint of the experiment after injected with 10^4^ CT26-Vector or CT26-CD45 cells (mice that have no metastasis were excluded for the calculation). All bar graph data are presented as means ± SEM. *P < 0.05, **P < 0.01 by Student’s *t* test or Mann-Whitney *U* test

As CTCs could internalize CD45 to evade immune surveillance in the circulation, we speculated that CD45^+^ CTCs could escape anti-cancer immunity and eventually colonize to distant organs. To test this hypothesis, CT26-Vector and CT26-CD45 cells were injected intracardially into immunodeficient Balb/C nude mice and immunocompetent Balb/C mice and their metastatic potential were evaluated. Consistent with our hypothesis, CT26-CD45 cells formed significantly more metastatic foci in lung tissues than CT26-Vector cells in immunocompetent Balb/C mice and the metastatic foci were clearly visualized by hematoxylin and eosin (H&E) staining (Fig. 6a–c). In contrast, there was no significant difference of lung metastatic foci formed by CT26-CD45 versus CT26-Vector cells in immunodeficient Balb/C nude mice. These results indicated that CD45^+^ CTCs could escape from immune surveillance and have a higher in vivo metastatic potential (Fig. 6d).

The number of CTCs in the circulation is extremely low. To better mimic the metastatic tumor formation of CTCs, we performed intracardiac injection of CT26-Vector or CT26-CD45 cells by limiting dilution (high gradient = 10^4^ cells, medium gradient = 10^3^ cells, and low gradient = 10^2^ cells) into Balb/C mice and non-invasively monitored metastasis formation weekly by In Vivo Imaging System (Fig. 6e). As expected, the high gradient groups ((both CT26-Vector and CT26-CD45) had the earliest visible metastasis formation and the highest metastatic rates (50–70%) (Fig. 6f). There was no observable metastasis formation by bioluminescence imaging among the low gradient group of CT26-Vector cells at the endpoint of the experiment (Fig. 6g). However, the medium and low gradient groups of CT26-CD45 cells formed more remarkable metastasis compared to the CT26-control groups, though the metastatic rates were low (25–30%) (Fig. 6h). Importantly, the high gradient group of CT26-CD45 cells progressed more rapidly and thus gave rise to shorter animal survival than the high gradient group of CT26-Vector cells when incorporating the mice that appeared metastasis before the endpoint of the experiment (Fig. 6i, j).

Together, these results demonstrated that CD45^+^ CTCs exhibited remarkably higher metastatic potential than CD45^−^ CTCs.

### Transferred CD45 attenuated immune surveillance to escort CTCs survival via diminishing TCR signaling

**Fig. 7:**
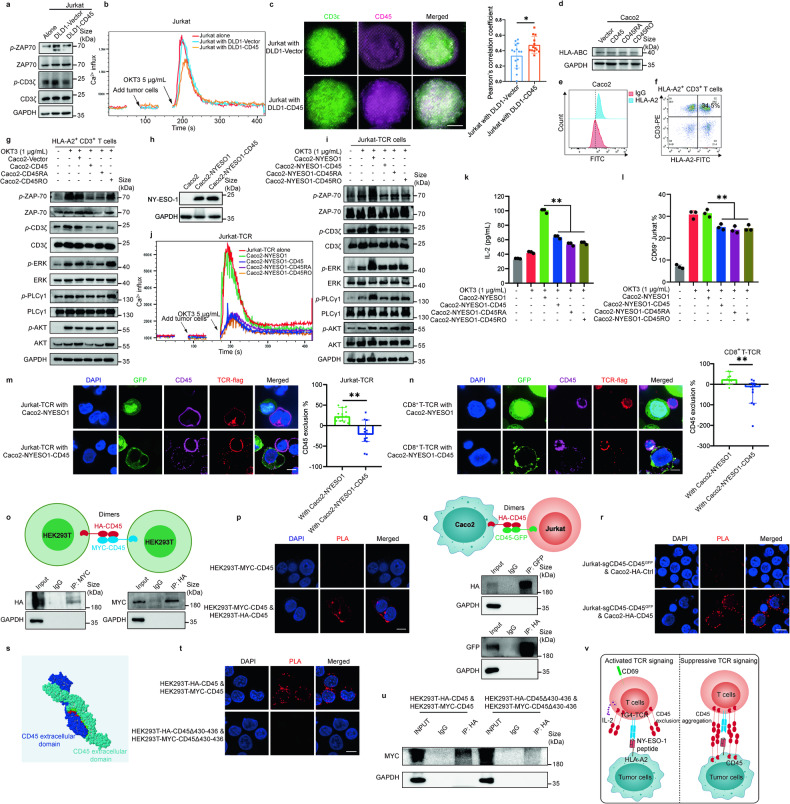
CD45 on tumor cells attenuates TCR signaling. **a** Immunoblot analysis for TCR signaling of Jurkat cells after activation with OKT3 (1 μg/mL) and cocultured with DLD1 cells overexpressing CD45 isoforms for 5 min. **b** Ca^2+^ influx of Jurkat cells after OKT3 (5 μg/mL) stimulation and cocultured with DLD1 cells with or without CD45 overexpression. **c** Immunofluorescence images of Jurkat cells after activation with OKT3 (1 μg/mL) and cocultured with DLD1 cells with or without CD45 overexpression for 5 min. Pearson’s correlation coefficient characterizes the colocalization of CD3ε (green) and CD45 (purple), each point represents one cell. Scale bar = 5 μm. **d** Immunoblot analysis for HLA-ABC expression of Caco2 cells overexpressing different CD45 isoforms. **e** Flow cytometric analysis of HLA-A2 expression on Caco2 cells. **f** Flow cytometric isolation of HLA-A2^+^ CD3^+^ T cells from PBMCs of healthy donors. **g** Immunoblot analysis for TCR signaling of HLA-A2^+^ CD3^+^ T cells after activation with OKT3 (1 μg/mL) and cocultured with Caco2 cells with or without CD45 overexpression within 5 min. **h** Immunoblot analysis for NY-ESO-1 expression in Caco2 cells after the indicated manipulation. **i** Immunoblot analysis for TCR signaling of Jurkat-TCR cells after activation with OKT3 (1 μg/mL) and cocultured with Caco2-NYESO1 cells with or without CD45 overexpression within 5 min. **j** Ca^2+^ influx of Jurkat-TCR cells after OKT3 (5 μg/mL) stimulation in the presence of Caco2-NYESO1 cells with or without overexpression of CD45 isoforms. **k** IL-2 secretion of Jurkat-TCR cells after cocultured with Caco2-NYESO1 cells with or without CD45 overexpression for 16 h. **l** Flow cytometric analysis of CD69 expression on Jurkat-TCR cells when cocultured with Caco2-NYESO1 cells with or without CD45 overexpression for 16 h. **m, n** CD45 exclusion from the immune synapse (IS) on Jurkat-TCR cells or CD8^+^ T-TCR cells in contact with Caco2-NYESO1 cells with or without CD45 overexpression after 5 min of activation with OKT3 (1 μg/mL), fixed, stained with anti-CD45 and anti-flag antibody. The exclusion percentage = (1-I_CD45 in IS zone_/I_CD45 out IS zone_) × 100%. I means intensity. Data are presented as median with 95% CI. Scale bar = 10 μm. **o** Schematic diagram showing the experimental setup for the investigation of the intercellular CD45-CD45 homophilic interactions. The co-immunoprecipitation data below illustrates the homophilic CD45-CD45 interactions in the cell model. **p** Immunofluorescence images showing the intercellular CD45-CD45 homophilic interactions detected by proximity ligation assay. Scale bar = 10 μm. **q** Schematic diagram showing the experimental setup for the investigation of the intercellular CD45-CD45 homophilic interactions. The co-immunoprecipitation data below illustrates the homophilic CD45-CD45 interactions between Caco2 cells overexpressing HA-CD45 and Jurkat-sgCD45-CD45^GFP^ cells. **r** Immunofluorescence images showing the intercellular CD45-CD45 homophilic interactions detected by proximity ligation assay. Scale bar = 10 μm. **s** Predicted crystal structure of CD45-CD45 interactions using ZDOCK. **t** The proximity ligation assay and (**u**) the co-immunoprecipitation data showing the homophilic CD45-CD45 interactions between HEK293T-HA-CD45 and HEK293T-MYC-CD45 cells with or without CD45 truncation of amino sites 430–436. Scale bar = 10 μm. **v** Schematic diagram showing the regulation of the TCR-pMHC complex and the potential immuno-modulatory role of CD45-CD45 dimerization between tumor cells and T cells. Unless otherwise specified, all bar graph data are presented as means ± SEM. *P < 0.05, **P < 0.01 by Student’s *t* test or Mann-Whitney *U* test

CD45 is pivotal in regulating T cell receptor (TCR) signaling.^25^ CD45 has enzymatic activity within its cytoplasmic phosphatase portion that is sufficient to inhibit TCR signaling of T cells at basal state.^26,27^ However, the extracellular domain of CD45 is longer than the TCR-pMHC complex, thus making it difficult to fit within the narrow contact interface between a T cell and an antigen presenting cell (APC).^28^ When TCR is engaged by its cognate MHC-bound peptide, CD45 is excluded from the immunological synapse (IS) to facilitate productive T cell activation.^29^ As we found that T cell activation was reduced when PBMCs were in co-culture with CD45-overexpressing tumor cells (Fig. 4), we speculated that CD45 on tumor cells could also play a suppressive function on TCR signaling of T cells (Supplementary Fig. 4a). To investigate the mechanism by which CD45 protect tumor cells from T cell killing, the phosphorylation levels of TCR signaling-related proteins was measured in Jurkat cells that were isolated from the co-culture system containing DLD1 cells. Decreased phosphorylation of CD3ζ and ZAP-70 (two important initiators of TCR signaling) was observed in Jurkat cells after coculturing with DLD1-CD45 cells for 5 min but not those coculturing with DLD1-Vector cells (Fig. 7a). The data indicated that CD45 expressing on the surface of tumor cells diminished TCR signaling to prevent T cell activation. Moreover, the calcium influx of Jurkat cells upon stimulatory anti-CD3 antibody (OKT3) shock was also detected in the presence of DLD1-Vector or DLD1-CD45 cells. The calcium influx of Jukat cells in the presence of DLD1-CD45 cells was significantly lower than that of Jurkat cells alone or in the presence of DLD1-Vector cells, implying the presence of DLD1-CD45 inhibited TCR signaling of Jurkat cells (Fig. 7b).

Antigen presentation and T cell activation require the exclusion of CD45 from the immune synapse to relieve phosphorylation of TCR signaling-initiating proteins such as lymphocyte-specific protein tyrosine kinase (Lck).^29–31^ To investigate the role of CD45 during this T cell activation process, we cocultured Jurkat cells with DLD1-Vector or DLD1-CD45 cells and stimulated T cell activation by OKT3. While CD45 on Jurkat cells showed minimal colocalization with CD3ε in co-culture with DLD1-Vector cells, CD45 was found to colocalize well with CD3ε in co-culture with DLD1-CD45 cells (Fig. 7c, Supplementary Fig. 4b). This finding suggested CD45 on tumor cells may prevent the formation of immune synapse.

The TCR-pMHC interaction is essential for the adaptive immune response and it is known to be an MHC-restricted process. T cells only respond to the antigen when it is bound to a particular MHC molecule. We firstly seek to exclude whether CD45 influences the MHC expression to influence TCR signaling. In Caco2-overexpressing CD45, CD45RA or CD45RO cells, the HLA-ABC expression remained unchanged (Fig. 7d). On the other hand, Caco2 cells were detected as an HLA-A2-restricted cells, which could be chosen to establish the specific TCR-pMHC ligation model (Fig. 7e). Next, HLA-A2^+^ CD3^+^ T cells were isolated from blood samples of healthy donors (Fig. 7f) and they were co-cultured with Caco2-Vector/Caco2-CD45/Caco2-CD45RA/Caco2-CD45RO cells. Phosphorylation status of a few critical TCR signaling-related proteins were examined. In HLA-A2^+^ CD3^+^ T cells coculturing with Caco2-CD45 or Caco2-CD45RA cells for 5 min (but not with Caco2-Vector cells), remarkable decrease in CD3ζ and ZAP-70 (as well as ERK and PLCγ1) phosphorylation was observed. On the other hand, the phosphorylation level of AKT, which was not an initiator of TCR signaling, remained unchanged. Interestingly, there was also minimal change in phosphorylation of the tested TCR signaling initiators when HLA-A2^+^ CD3^+^ T cells were cocultured with Caco2-CD45RO compared to those with Caco2-Vector cells. The discrepancies in the effect of the three CD45 isoforms may be attributed to the differences between their extracellular domain (Fig. 7g).

In addition, we exogenously introduced an HLA-A2-restricted TCR (1G4-TCR) that specifically recognizes the NY-ESO-1 peptide (SLLMWITQC) on Jurkat cells (hereafter abbreviated as Jurkat-TCR cells) and ectopically overexpressed NY-ESO-1 in HLA-A2-restricted Caco2 cells (hereafter abbreviated as Caco2-NYESO1 cells) (Fig. 7h). Consistently, the phosphorylation levels of CD3ζ, ZAP-70, ERK, and PLCγ1 relating to TCR signaling in Jurkat-TCR cells were remarkably increased when cocultured with Caco2-NYESO1 cells in the presence of OKT3 for 5 min. However, phosphorylation of the same TCR signaling initiators were suppressed when cocultured with Caco2-NYESO1-CD45 cells, regardless of CD45 isoforms (Fig. 7i). Furthermore, upon OKT3 shock, the calcium influx of Jurkat-TCR cells under OKT3 shock in the presence of Caco2-NYESO1 cells overexpressing CD45 isoforms was found to be significantly decreased. On the other hand, Jurkat-TCR cells alone or co-culture of Jurkat-TCR and Caco2-NYESO1 cells exhibited similarly high levels of calcium influx (Fig. 7j). In addition, IL-2 secretion and CD69 expression of Jurkat-TCR cells were decreased when they were cocultured with Caco2-NYESO1-CD45 cells compared to those cocultured with Caco2-NYESO1 cells (Fig. 7k, l). The possibility of CD45 exclusion from the immune synapse on Jurkat-TCR cells was also evaluated in co-culture of Jurkat-TCR and tumor cells. As indicated by the immunofluorescence staining data, CD45 was shown to be excluded from the immune synapse of Jurkat-TCR cells with Caco2-NYESO1 cells, but not from the immune synapse of Jurkat-TCR cells in co-culture with Caco2-NYESO1-CD45 cells (Fig. 7m). Similarly, CD45 exclusion on CD8^+^ T cells overexpressing 1G4-TCR was also confirmed (Fig. 7n).

CD45 can exist in the dimeric form and it has been reported that dimerization of CD45 down-regulates TCR signaling.^32,33^ We speculated whether intercellular CD45-CD45 homophilic interactions (i.e., dimerization) may occur in co-culture of CD45-shieded tumor cells and T cells. To this end, when HEK293T cells overexpressing HA-CD45 or MYC-CD45 were cocultured at 1:1 ratio for 3 h, the HA-CD45 and MYC-CD45 homophilic interactions were demonstrated by co-immunoprecipitation assay and proximity ligation assay (PLA) (Fig. 7o, p, Supplementary Fig. 4c, d). Similar results were confirmed when Jurkat-sgCD45 cells rescuing CD45 overexpression with CD45-GFP fusion (Jurkat-sgCD45-CD45^GFP^) were cocultured with Caco2 cells overexpressing HA-CD45 (Caco2-HA-CD45) at 1:1 ratio for 3 h (Fig. 7q, r). However, when Jurkat-sgCD45-CD45^GFP^ cells were cocultured with Caco2-HA-Ctrl cells, the PLA fluorescence intensity was significantly weaker than those cocultured with Caco2-HA-CD45 cells (Fig. 7r). In addition, rigid protein–protein docking (ZDOCK) was performed with two same human CD45 extracellular regions, domains d1-d4 to study their dimerization. The ZDOCK score values and their best pose interactions were calculated (Supplementary Table 5). The ZDOCK score of the two human CD45 extracellular regions, domains d1-d4 was 1314.717. Comprehensive analysis revealed that they formed hydrogen bond links with amino acid sites such as LYS430-LYS430, ASN524-THR333, thus can establish a stable protein docking model (Supplementary Fig. 7s). Moreover, the amino acid sites from 430–436 were predicted highly correlated with the dimerization, thus we truncated this region and established HEK293T-HA-CD45Δ430-436 cells and HEK293T-MYC-CD45Δ430-436 cells. Interestingly, their homophilic interactions from the two cells were significantly decreased as indicated by PLA and co-immunoprecipitation assay (Fig. 7t, u).

Based on “kinetic segregation” and “TCR-pMHC ligation” model, we hypothesized that the intercellular CD45-CD45 dimerization could protect tumor cells from T cell recognition and killing by preventing CD45 exclusion from the MHC I-peptide-TCR complex^34,35^ (Fig. 7v). This may explain why CD45 expression on tumor cells could suppress TCR signaling and allow tumor cells to escape from immune surveillance.

## Discussion

CTCs are well-known tumor metastasis precursors, which represent important biomarkers for estimating tumor progression and predicting prognosis of cancer patients.^2^ There has been extensive research in recent years to investigate the characteristics and mechanisms by which CTCs survive in the circulation and colonize distant organs. CTCs are isolated from the bloodstream and they are usually sorted by the epithelial cell adhesion marker EpCAM. On the other hand, CD45 has been used to target the surface of noncancerous cells for negative enrichment during CTC isolation.^36^ In this study, we identified a subpopulation of CTCs expressed CD45 (CD45^+^ CTCs) in blood samples from cancer patients with CRC, NSCLC or HCC. Importantly, single-cell CNA sequencing confirmed the malignant phenotype of these CD45^+^ cells. It is noteworthy that CD45^+^ CTCs were more closely correlated with metastasis and poor prognosis than CD45^−^ CTCs. We aimed to investigate whether CD45 enables CTCs to escape from immune surveillance and survive in the blood circulation to metastasize in distant organs.

Extracellular vesicles have been shown to facilitate the intercellular transfer of pro-tumorigenic factors in the tumor microenvironment.^37^ They promote angiogenesis, invasion, and proliferation in recipient cells to support tumor growth and pro-metastatic phenotype. When tumor cell EVs carry immune-modulatory proteins, these proteins may interact with specific receptors on immune cells to suppress immune cells.^16,17^ On the other hand, EVs secreted by immune cells were previously thought to exhibit anti-tumor properties.^18,38^ More recently, accumulating evidence suggest that immune cell-derived EVs may have pro-tumor effects.^39,40^ For instance, EVs-contained FasL derived from activated T cells was shown to promote melanoma evasion via the Fas/FasL pathway.^39^ However, the precise mechanisms remain elusive.

CD45 is a leucocyte common antigen that is expressed on almost all nucleated blood cells but not on tumor cells. Intriguingly, we first demonstrated that CD45 encapsulated in WBC-derived EVs could be transferred to the surface of tumor cells (Fig. 3). Although CTCs fused with macrophage cells has been shown to possess both epithelial and mesenchymal phenotypes, it cannot explain why small size CD45^+^ CTCs (< 10 μm) were found in cancer patients.^8^ Our finding about the transfer of EVs-derived CD45 from WBCs to CTCs may shed light on this question. In addition, it has been reported that the bone microenvironment could invigorate metastatic seeds (i.e., CTCs) for further dissemination.^41^ CTCs may come into close contact with hematopoietic stem/progenitor cells in the bone marrow, and undergo genetic and epigenetic alterations to escape from immune attack. Indeed, CTCs have been reported to hijack immune molecules to evade immune surveillance and promote the metastatic seeding processes.^7,42^ Nevertheless, more research is needed to elucidate the major nucleated blood cell subtype(s) that produce the EVs-derived CD45. Moreover, it is also important to investigate the critical modulator(s) that regulate the secretion of EVs-derived CD45 from blood cells and their subsequent internalization by tumor cells. Lastly, CTCs that survive in the circulation will eventually penetrate the vascular endothelium and colonize into appropriate niche. Our data showed that CT26-CD45 cells grew significantly faster than CT26-Vector cells in immunocompetent mice but not in immunodeficient mice (Fig. 5). Therefore, the expression of CD45 in tumor cells may suppress immune responses and orchestrate an immunosuppressive microenvironment in vivo. This postulate was further substantiated by data obtained in our in vivo metastasis study where CD45^+^ CTCs exhibited significantly stronger metastatic potential than CD45^−^ CTCs (Fig. 6). Importantly, CD45^+^ CTCs were also shown higher metastatic potential than control CTCs despite extremely low CD45^+^ CT26 cell numbers (Fig. 6h).

The tyrosine phosphatase CD45 lacking transmembrane and extracellular domains has been reported to regulate TCR signaling via its phosphorylase domain activity.^43^ It has also suggested that the dimeric form of CD45 could down-regulate TCR signaling.^32,33^ In our study, we demonstrated the intercellular CD45-CD45 homophilic interactions in co-culture of HEK293T cells independently overexpressing HA-CD45 and MYC-CD45 (Fig. 7o–u). Therefore, we proposed that CD45-CD45 dimers may be formed between tumor cells shielded with CD45 and T cells that anchored CD45 in the immune synapse to inhibit ligated TCR signaling via its phosphatase activity though further strong evidence is needed to confirm this model (Fig. 7v). Consistently, in our HLA-A2 restricted TCR-pMHC system (Fig. 7g–n), CD45-overexpressing tumor cells were shown to cause CD45 aggregation in immune synapse to suppress the activation of TCR signaling of T cells. Therefore, tumor cells overexpressing CD45 were found to be resistant to T cell killing and T cell secretion of cytotoxic factors such as IFN-γ was significantly decreased (Fig. 4). To this end, the large ectodomains of CD45 are known to be heavily O-glycosylated and N-glycosylated and that the changes in CD45 glycosylation could regulate CD45 segregation from the immune synapse and inhibit TCR signaling.^26,44^ It is still not clear whether and how differential glycosylation of CD45 extracellular domain, or variation in dimerization of different CD45 isoforms influence the TCR-pMHC ligation and TCR signaling transduction. Interestingly, overexpression of the shortest isoform of CD45, CD45RO in tumor cells were shown to bidirectionally regulate TCR signaling. Thus, size-dependent TCR-pMHC ligation may also be involved in CD45-regulated T cell activation.^35^ Furthermore, the intracellular domain of CD45 has tyrosine phosphatase activity. Park et al. reported that CD45 overexpression enhanced stemness and therapy-resistant phenotype of colorectal cancer cells, as a result of its phosphatase activity promoting Wnt transcription by dephosphorylating and stabilizing the β-catenin protein.^45^ In addition, the extracellular domain of CD45 has complex interactions with other molecules such as BTN3A1 and galectin-1 that can be secreted in the circulation.^46,47^ These interactions may change the phosphatase activity of CD45, thus regulating the downstream signaling. Further studies are needed to focus on its specific ligand(s), which may play pivotal role in regulating CD45 function.

In summary, our study is the first to demonstrate that CD45^+^ CTCs, an unheeded subtype of CTCs which acquire their CD45 expression from nucleated blood cell-derived EVs, are common among cancer patients. CD45^+^ CTCs are closely correlated with metastasis and worse prognosis than CD45^−^ CTCs. When shielded by CD45, CTCs can easily evade immune surveillance and resist T cell killing by intercellular CD45-CD45 homophilic interactions between CTCs and T cells that diminish TCR-signaling. Our results thus provide a rationale for targeting EVs-derived CD45 internalization by CTCs to prevent cancer metastasis.

## Materials and Methods

### Ethics approval and consent to participate

Animal study (L102012021110J) was performed with the permission of the institutional committee of Sun Yat-sen University Cancer Center, in compliance with protocols approved by the Guangdong Provincial Animal Care and Use Committee and experimental guidelines of the Animal Experimentation Ethics Committee of Sun Yat-sen University Cancer Center. All blood specimens were obtained at the Sun Yat-sen University Cancer Center under the study protocols SL-G2022-020-01, approved by ethics committee of Sun Yat-sen University Cancer Center.

### Patients

CRC patients who were diagnosed for the first time or who developed progressive disease after extensive treatment; or randomly selected NSCLC, HCC as well as BC patients were recruited for the study. The study protocol SL-G2022-020-01 was approved by the research ethics committee of Sun Yat-sen University Cancer Center. After obtaining written informed consent from the subjects, peripheral blood samples (5–10 mL) were drawn at the Sun Yat-sen University Cancer Center in EDTA vacutainers. All blood specimens were obtained at the Sun Yat-sen University Cancer Center under the study protocols SL-G2022-020-01, approved by ethics committee of Sun Yat-sen University Cancer Center. Patients who were affected by two or more tumor types at the same time were excluded from the study.

### Cell culture

Human colorectal cancer cell lines (Caco2, DLD1, THC8307), mouse colorectal cancer cell line (CT26), human leukemia cell lines (Jurkat, THP1) were obtained from the American Type Culture Collection, ATCC. They were grown in RPMI 1640 medium (C11875500BT, GIBICO) supplemented with 10% fetal bovine serum (03-033-1BCS, BI), 100 U/mL penicillin (H44022447, TIANXIN), 100 U/mL streptomycin (H37020187, LKPC) in a humidified incubator at 37 °C with 5% CO_2_. Peripheral blood monocytes (PBMCs) isolated from healthy donors using PBMC Isolation Kit (P8610, Solarbio) were cultured in the above complete medium supplemented with 150 U/mL IL-2. HEK293T and Raw264.7 cells were maintained in DMEM medium (C11995500BT, GIBICO) supplemented with 10% fetal bovine serum, 100 U/mL penicillin, and 100 U/mL streptomycin in a humidified incubator at 37 °C with 5% CO_2_.

### CTC capture

Human CTCs were isolated from unprocessed peripheral blood samples within 2 h of blood collection according to the procedure recommended for using the Cellab Thomas I CTCs processing workstation. Briefly, 5–10 mL EDTA-anticoagulated blood samples obtained from cancer patients were centrifuged at 500 g for 10 min to remove plasma. Then, an equivalent volume of CTCs preservation solution was gently added to the blood tubes and uniformly mixed. CTCs were separated and enriched from the blood samples within 20 min. The isolated CTCs were used directly in biological assays or maintained in culture.

### CTCs capturing using NextCTC and downstream CEP8 staining

Fresh blood samples from cancer patients were immediately performed RBC lysis for 5 min at room temperature. After harvesting cell pellets by 1300 rpm centrifugation for 5 min, they were resuspended with PBS and used for CTCs isolation using NextCTC CTC-capturing apparatus from FOCUSGEN. CTC solutions were centrifugated at 1300 rpm for 10 min to conserve CTCs solutions 100 μL. The solutions were prefixation with CF1 1–2 µL and used for cell smear on the glass slides. After making sure the glass slides were dry at 50 °C, the slides underwent fixation with CF2 100 µL for 7 min, aging at 37 °C for 30 min, dehydration in 75% ethyl alcohol for 1 min and absolute ethyl alcohol for 1 min. Then CEP8 probe reagent 10 µL was added onto the slides and performing probe hybridization in the PCR amplifier at 75 °C/12 min, 37 °C/4 h. The slides were successively washed in solution A twice for 2 min and solution B at 60–68 °C for 3 min, then washed in solution A for 2 min and in PBS twice for 2 min. Once the slides were dry, FITC-CD45 antibody was added with a dilution ratio 1:100 at 4 °C overnight in the dark place. After washed with PBS twice for 2 min and added DAPI for 10 min, another twice washing with PBS for 2 min was necessary for the next fluorescence observation. The red CEP8 phosphor dots ≥ 3 in the cell nucleus was considered malignant. The CTCs-FISH Kit (including CEP8, FITC-CD45 and other buffer solutions used here) is from FOCUSGEN.

### CTC short-period culture in vitro

FACS sorted CTCs were grown in ultralow attachment 96-well round bottom plates (7007, Corning) containing CTC culture medium. The medium is composed of RPMI-1640 medium and a series of growth factors and inhibitors including EGF (20 ng/mL, 78006.2, Stemcell), bFGF (20 ng/mL, 78003.2, Stemcell), HGF (20 ng/mL, 78019.2, Stemcell), Y-27632 (10 μM, S1049, Selleck), Acetylcysteine (1.25 mM, T0875, TargetMol), L-glutamine (200 mM, T0326L, TargetMol), N-2 Supplement (0.5×, 17502048, ThermoFisher) and B27 (0.5×,17504044, GIBCO). For quantifying the change of CD45^+^ CTCs percentage, sorted cells were divides into two tubes. One is immediately fixed with 4% PFA for later immunofluorescence staining and another is transferred into the CTC culture wells and cultured in a humid 37 °C incubator with 5% CO_2_ and 4% O2 for 72 h. After that, both 4% PFA-fixed cells were used for immunofluorescence staining.

### Extracellular vesicles isolation

Jurkat and THP1 cells were cultured in RPMI 1640 medium containing 1% EVs-depleted FBS at a density of 2 million cells per mL medium. HEK293T cells overexpressed CD45-GFP fusions or Raw264.7 cells were cultured in DMEM medium containing 10% FBS up to a confluency of about 80%. Then, the culture medium was replaced with DMEM medium containing 1% EVs-depleted FBS. Tumor cell-derived conditioned medium was collected after 48 h of culture, and EVs were isolated by differential centrifugation using the EVs isolation protocol as described previously.^48^ Briefly, after removing cells and debris by centrifugation at 500 g for 5 min and filtration with 0.45 um sterile filters (SLHVR33RB, Millipore), the supernatant was harvested and centrifuged at 15,000 g for 45 min to remove large extracellular vesicles. Finally, the supernatant was centrifuged at 100,000 g for 90 min (All ultracentrifugation steps were performed using Beckman Coulter Avanti J30I at 4 °C). Sedimented EVs were re-suspended and washed in PBS followed by another ultracentrifugation procedure (100,000 g, 90 min) and the pellet was resuspended in 200 μL PBS.

For sucrose density gradient centrifugation, the pellet collected from ultracentrifugation (100 μg) was overlaid with a linear sucrose density gradient (10–70% w/v, pH 7.4) created by a gradient fractionator (Biocomp YIQI 113) in a SW41 tube (Beckman Coulter). The gradients were subjected to ultracentrifugation (Beckman Coulter Optima^TM^ L-100 XP) at 100,000 g for 16 h at 4 °C. Gradient fractions (1 mL) were collected from top to bottom and then the fractions were washed in PBS followed by ultracentrifugation (Beckman Coulter Optima^TM^ MAX-XP) at 100,000 g at 4 °C for 2 h. Sediments from the fractions were directly lysed in RIPA buffer for further immunoblot analysis.

For plasma EVs isolation, EDTA-anticoagulated blood samples (3–5 mL) obtained from cancer patients were firstly centrifuged with 500 g for 10 min to harvest about 1 mL of plasma, they were then diluted with equal volume of PBS, filtered, and ultracentrifuged (Beckman Coulter Optima^TM^ MAX-XP) sequentially at 15,000 g at 4 °C for 1 h and 100,000 g at 4 °C for 2 h. The sediments were resuspended in 100 uL PBS for further immunoblotting analysis, nano-flow cytometry and ELISA assay.

### Nanoparticle tracking analysis

The Nanosight NS300 system (Nanosight Technology, Malvern, UK) was used to analyze the number and size distribution of fresh EVs according to manufacturer’s instructions.

### Transmission electron microscope imaging

Fresh EVs collected from supernatant of 10 million Jurkat cells were resuspended in 50 μL ice-cold PBS and were negatively stained with 2% uranyl acetate solution and imaged by the Tecnai G2 SpiritTwin electron microscope (FEI).

### Immunofluorescence staining

For CTCs staining, CTC suspension was centrifuged at 250 g for 5 min to concentrate the CTCs into 50 uL suspension, which was then precipitated for 15 min on poly-lysine precoated well of 96-well plate (165305, Thermo Fisher). Cells were fixed in 4% paraformaldehyde for 15 min and permeabilized for 10 min in 0.2% Triton-X100. Following 1 h blocking with 4% bovine serum albumin, the CTC specimens were co-stained with pan-cytokeratin monoclonal antibody conjugated with Alexa Flour^TM^ 488 (53-9003-82, Thermo Fisher), PE mouse anti-human CD45 (555483, BD Pharmingen^TM)^, anti-CD45 (ab40763, Abcam) antibody, FITC anti-human CD326 (EpCAM, FHF326-01-100, 4Abio), CoraLite488-conjugated Vimentin mouse monoclonal antibody (CL488-60330, Proteintech), anti-HER2 (18299-1-AP, Proteintech) antibody and DAPI (D001, MDbio). The secondary antibody used for detecting CD45 and HER2 are anti-rabbit IgG-AF568 (A11011, Invitrogen) and anti-rabbit IgG-AF488 (A32790, Invitrogen), respectively. For adherent cells, cells were precultured with different conditions on the 35 mm confocal plate and then they were fixed following the staining procedure described above. The images were captured and analyzed by confocal laser microscope LSM880, confocal living cell imager Nikon CSU-W1, or fluorescence microscope Nikon ECLIPSE Ti2. To visualize the colocalization of CD45 and CD3ε (ab16669, Abcam), super-resolution microscope N-SIM/N-STORM was also used.

### T cells mediated tumor killing

The tumor cells used for T cells killing included: (i) CD45^+^ DLD1 and CD45^−^ DLD1 cells sorted from parent DLD1 cells after previous indirect coculture with Jurkat cells; (ii) DLD1 cells pre-incubated with Jurkat cell-derived EVs for 12 h to acquire CD45 expression; (iii & iv) DLD1 and Caco2 cells overexpressing CD45 or not, separately. They were plated at a density of 25,000 cells per well in 24-well plates and then co-cultured with PBMCs at PBMC-to-tumor cell ratios (E:T) of 0:1, 1:1, 2:1, or 4:1 for at least 72 h until the cell density of the control group reached 90%. Afterwards, the wells were washed with PBS twice to remove PBMCs and the adherent and surviving tumor cells were fixed with 4% paraformaldehyde and stained with 0.05% crystal violet solution. Colorimetric analysis was used to determine the viability of tumor cells.

### NK cell killing assay

NK cells were sorted from 10 mL peripheral blood of heathy donors. In short, WBCs were harvested by RBC lysis for 5 min at room temperature. After washed with PBS twice and diluted with 2% FBS PBS, the following antibody were added to the solution and kept on ice in dark for 30 min: (1) PE anti-human CD45 (304008, Biolegend); (2) PerCP anti-human CD3 (317337, Biolegend); (3) FITC anti-human CD56 (NCAM) (318303, Biolegend). CD45^+^ CD3^−^ CD56^+^ NK cells were sorted using Overspeed flow cytometer for cell sorting (MoFlo Astrios, Beckman-Coulter). Before NK cell killing of cancer cells, Caco2 cells were stained with Calcein Live Cell staining Kit (CA1630-500T, Solarbio) for 30 min at 37 °C. The cell culture medium used for the NK cell killing assay was RPMI-1640 containing 10% FBS, 1000 U/mL IL-2, 10 ng/mL IL-15 (200-15-2, PeproTech). NK cells were cocultured with calcein-stained Caco2 cells (with effector-to-target ratios of 80:8, 40:8, 20:8, 10:8, 5:8) in U-type 96-well plates for 4 h. Then the plates were centrifuged at 100 g for 5 min, 100 μL medium from each well was carefully transferred into new 96-well plates and the released calcein-fluorescence from each well was measured by TECAN multifunctional enzyme labeling instrument (Spark 10 M). The percentages of lysis were calculated and presented as: (R_test_ - R_auto_) / (R_max_ - R_auto_) × 100% (R_max_ means released calcein-fluorescence from wells of calcein-stained Caco2 cells treated with 2%Triton-X100, R_auto_ means released calcein-fluorescence from wells of calcein-stained Caco2 cells without any treatment).

### Constructs

The C-terminus CD45-GFP, N-terminus HA-CD45 and MYC-CD45 fusion constructs were generated by ligating the CD45 coding sequence (NM_002838.5) with a GFP, HA, and MYC tag, respectively, into the pLVX-MCS-Puro lentiviral expression vector using standard molecular cloning techniques. Exons 5 and 6 of *PTPRC* were removed to generate CD45RA whereas exons 4, 5 and 6 of *PTPRC* were removed to produce CD45RO according to the manufacturer’s protocol. In addition, truncated HA-CD45Δ430-436 and MYC-CD45Δ430-436 were also produced. Besides, *CTAG1B* encoding NY-ESO-1 was ligated into pLVX-IRES-Neo. CRISPR RNA (crRNA) targeting *PTPRC* (Forward primer: CCAAATGGTAACGTTCATGG), *Ptprc* (Forward primer: ATACTATTGTCTGTCGGCC), *Rab27a* (Forward primer: GTACTCTACCAGTACACTGA) and a negative control (NC) sequence (Forward primer: CGCGATAGCGCGAATATATT) were constructed into the lentiviral transfer plasmid CRISPR/Cas9 LentiCRISPR V2. pLKO.1-puro was used to mediate shRNA expression of *RAB27A* (Forward primer: CCAGTGTACTTTACCAATATA). These lentiviral constructs were co-transfected with psPAX2 and pMD2.G at a ratio of 4:3:1 into HEK293T cells using Lipofectamine 2000 (11668019, Thermo Fisher) in Opti-MEM medium. The total DNA-to-Lipofectamine 2000 ratio was maintained at a ratio of 1:2 during the transfection. After 6 h of transfection, the medium was replaced with fresh DMEM and the supernatants containing viral particles were then collected from the cells after 48 h. The supernatants were then subjected to filtration using 0.45 μm PES filter and were used to transfect cancer cells with polybrene (8 μg/mL) for 48 h. Stably transfected cells were maintained in either puromycin dihydrochloride (5 μg/mL, 0219453925, MP) or G418 sulfate (500 μg/mL, ST081, Beyotime) and expression of protein of interest was confirmed by immunoblotting. Lentiviral vector was used for transduction of suspension cells using the spinfection protocol. The sequence of HLA-A2-restricted TCR (1G4-TCR) that specifically recognizes NY-ESO-1 (SLLMWITQC) was synthesized and cloned into a 3× FLAG-tag bearing pLVX-MCS-Puro. Jurkat cells were spinfected (2000 g for 90 min, 25 °C) in virus-containing medium with 8 μg/mL polybrene for transfection. The medium was replaced with fresh medium after transfection for 24 h. The sequence of the constructs was confirmed by GUANGZHOU RUIBIO TECHNOLOGY.

### Immunoblotting

Protein lysates from freshly collected tumor cells or frozen tumor tissues were generated using RIPA buffer supplemented with protease inhibitor cocktail (C0001, TargetMol). The protein concentrations in lysates were determined using BCA Protein Assay Kit (XYM-THE-23227, biosharp). The lysates were electrophoresed in 8–12% SDS-PAGE gel and the separated proteins were transferred to a polyvinylidene difluoride (PVDF) membrane (IPVH00010, Millipore) for antibody incubation. After blocking with 5% skimmed milk, PVDF membranes were blotted with primary antibodies (1:1,000-1:2,000 dilution) overnight at 4 °C and secondary antibody (1:10,000 dilution) for 1 h at room temperature. Protein bands were visualized by ECL imaging (Clarity Western ECL Substrate, 1705061, Bio-Rad) using Bio-Rad ChemiDoc Imaging System. Detailed antibody information was listed as following: CD45 (60287-1-Ig, Proteintech), CD63 (ab59479, Abcam), GAPDH (60004-1-Ig, Proteintech), TSG101 (DF8427, Affinity), Calnexin (AF5362, Affinity), CD31 (11265-1-AP, Proteintech), CD9 (20597-1-AP, Proteintech), RAB27A (DF6702, Affinity), GFP tag (50430-2-AP, Proteintech), ZAP-70 (AF8390, Beyotime), Phospho-ZAP70 (Tyr319) (*p*-ZAP70, AF5968, Beyotime), CD3ζ (AF0096, Beyotime), Phospho-CD3ζ (Tyr142) (*p*-CD3ζ, AF5425, Affinity Biosciences), ERK1/2 (ERK, 51068-1-AP, Proteintech), Phospho-ERK1/2 (Thr202/Tyr204) (*p*-ERK, 28733-1-AP, Proteintech), AKT (60203-2-Ig, Proteintech), Phospho-AKT (Ser473) (*p*-AKT, 66444-1-Ig, Proteintech), PLCγ1 (2822 S, CST), Phospho-PLCγ1 (Tyr783) (*p*-PLCγ1, 2821 S, CST), NY-ESO-1(19521-1-AP, Proteintech), HLA-ABC (15240-1-AP, Proteintech), HA tag (51064-2-AP, Proteintech), MYC tag (16286-1-AP, Proteintech).

### Co-immunoprecipitation

HEK293T cells overexpressing MYC-CD45 or HA-CD45 (as well as HEK293T-HA-CD45Δ430-436 cells and HEK293T-MYC-CD45Δ430-436 cells) were cocultured at a 1:1 ratio in the ultra-low attachment culture plate for 3 h. The cells were then harvested and lysed by NP40 lysis buffer supplemented with protease inhibitor cocktail (C0001, TargetMol). After centrifugation at 140,000 g for 10 min at 4 °C, the supernatants were collected and incubated with HA-tag Rabbit antibody (51064-2-AP, Proteintech) 10 μg/mL or MYC-tag Rabbit antibody (16286-1-AP, Proteintech) 15 μg/mL overnight at 4 °C. Protein A/G Magnetic Beads (B23202, Bimake) were used for the immunoprecipitation (1 h incubation at room temperature) to pull down the IgG-bound proteins. The immunoprecipitated protein samples were further analyzed by immunoblotting. Similarly, Jurkat-sgCD45-CD45^GFP^ and Caco2-HA-CD45 cells were also cocultured to perform the co-immunoprecipitation assay.

### Flow cytometry

Anti-human or anti-mouse antibodies were fluorescence-conjugated. Carboxyfluorescein succinimidyl ester (CFSE) cell labelling kit (HY-D0938, MCE) was used for the fluorescent intracellular labelling of live cells. Briefly, freshly collected cells were washed twice and then incubated with desired antibody in 100 μL PBS on ice for 20 min. The samples were then washed twice and resuspended in 200 μL PBS for data acquisition using a cytoFLEX flow cytometer within 2 h. To detect the T cell activation marker CD69, Jurkat-TCR cells were cocultured with Caco2-NYESO1 cells or Caco2-NYESO1-CD45 cells for 24 h, CD69 expression in the Jurkat-TCR cells was analyzed by using FITC conjugated anti-human CD69 antibody (310904, Biolegend).

For nano-flow cytometry, EVs were resuspended in 100 μL PBS (0.1 μm filter filtration) and added 4 μL FITC anti-human CD9 (312103, Biolegend), 4 μL APC anti-human CD45 (304012, Biolegend), 4 μL PE anti-human CD61 (336406, Biolegend). All these antibodies were centrifuged at 17,000 g for 30 min to exclude impurity effect before adding. After incubating at 4 °C for 1 h, the samples were diluted with 300 μL PBS (0.1 μm filter filtration). Nano-flow cytometry analysis of EVs were performed according to the protocol of the CytoFLEX flow analyzer with 405 nm laser.

Other antibodies and regents used for flow cytometry analysis were listed as following: Rabbit IgG Fluorescein-conjugated Antibody (F0112, R&D), Mouse IgG1 PE-conjugated Antibody (IC002P, R&D), FITC anti-human HLA-A2 (343303, Biolegend), PE anti-human CD3 (12-0039-42, eBioscience), PKH 26 Red Fluorescent Cell Linker Mini Kit (MINI26, Sigma-Aldrich), IFN-γ (CI57-10, NovoProtein), 30% H_2_O_2_ (66005, MDbio), Chlorpromazine (C424348, Aladdin).

### ELISA

CD45 expression of EVs harvested from plasma or supernatant of Jurkat-Ctrl cells, Jurkat-sgCD45 cells, Jurkat-shRAB27A cells were measured by the Human CD45 ELISA Kit (DG12518H, Dogesce). To assess the cytotoxicity of T cells, the release of IFN-γ (1110002, DAKEWE), TNF-α (1117202, DAKEWE), and IL-2 (1110202, DAKEWE) was detected in the supernatants of PBMCs after 16 h co-culture incubation with tumor cells at a 4:1 ratio.

### qRT-PCR

RNA extraction from interested cells using EZ-press RNA Purification Kit (B0004D, EZBioscience). Ensure proper handling and storage of RNA to maintain its integrity. Convert the extracted RNA into complementary DNA (cDNA) using a reverse transcription reaction using Color Reverse Transcription Kit (with gDNA Remover) (A0010CGQ, EZBioscience). Prepare the PCR reaction mixture containing the cDNA template, gene-specific primers (*GAPDH* Forward: TTCTTTTGCGTCGCCAGCC, *PTPRC* Forward: GACACGGCTGACTTCCAGAT) and Color qPCR reagent kit (A0012-R1, EZBioscience). Perform the PCR amplification using qPCR ROCHE 480 instrument. The qRT-PCR data was analyzed by GraphPad Prism 8 using Student’s *t* test.

### Cancer cell proliferation assay

For proliferation assay, DLD1-Vector, DLD1-CD45, DLD1-CD45RA, DLD1-CD45RO cells (3000 cells each) were seeded into 96-well plates and cultured with RPMI 1640 containing 10% FBS. After different duration of cell growth (1, 3, 5, and 7 days), 20 μL MTT solution (5 mg/mL) was added into each well of the different groups and incubated for 4 h at 37 °C. MTT crystal was dissolved in 100 uL DMSO after discarding the supernatant from each well. Cell proliferation was then assessed by measuring the optical density at 570 nm (630 nm as reference wavelength) using a microplate reader (Bio-Rad). The experiments were carried out three times.

### Ca^2+^ influx assay

In brief, Jurkat cells (3 million cells) were washed and resuspended in 500 μL dilution buffer containing Fluo-8^AM^ and Pluronic F127 (C0012, APPLYGEN) for a 30 min incubation at room temperature. DLD1 or Caco2 overexpressing CD45 were stained with PKH 26 at room temperature, washed, resuspended in dilution buffer, and placed on ice. Jurkat cells were then washed and resuspended at a density of 3 million cells per mL in dilution buffer The Ca^2+^ influx of Jurkat cells alone or in the presence of equal volume of tumor cells (half the number of Jurkat cells) with or without the anti-CD3 antibody (OKT3) 5 μg/mL shock was analyzed within 7 min using an ACEA NovoCyte flow cytometry.

### Proximity ligation assay

HEK293T cells overexpressing MYC-CD45 or HA-CD45 (Another group is Jurkat-sgCD45-CD45^GFP^ and Caco2-HA-CD45 cells) were cocultured at a 1:1 ratio in the ultra-low attachment culture plate for 3 h. Then they were harvested for fixation and permeabilization. After incubation in blocking buffer at 37 °C for 1 h, the cells were incubated with HA-tag mouse antibody (RM1004, Ray antibody) 1:50 and MYC-tag rabbit antibody (16286-1-AP, Proteintech) 1:50. The proximity ligation assay was performed using Duolink® In Situ Detection Regents Red (DUO92008, Sigmaaldrich) according to the manufacturer’s instructions. Fluorescent images indicating protein-protein interactions were captured by the Zeiss LSM880 confocal laser scanning microscope.

### Establishment of in vivo tumor xenograft and metastatic tumor models

All experiments were conducted according to the protocol approved by Sun Yat-Sen University Institutional Animal Care and Use Committee (L102012021110J). Immunodeficient Balb/C nude or immunocompetent Balb/C female mice (4-week-old) were obtained from GDMLAC or Gempharmatech-GD for all experiments. Balb/C or Balb/C nude mice were implanted with tumor xenografts by subcutaneously injecting 5 × 10^5^ CT26 cells into the bilateral flank. For one group, the left flank is implanted with CT26-Vector cells whereas the right flank is implanted with CT26-CD45 cells. For the other group, both flanks were implanted with CT26-Vector cells. Equal volumes of PBS and Raw 264.7-derived EVs (120 μg/mL, 3 μg/mouse) were injected into the tumors in the left and right flank every 3 days. The tumor size was assessed every 3 days with electronic calipers. When the maximum tumor diameter reached 15 mm, the experiment was terminated. The tumor volume was calculated using the formula: tumor volume = length × width^2^ / 2. For unilateral subcutaneous tumor xenografts, 6 × 10^5^ CT26 cells were used. And for each group, equal volumes of PBS or Raw 264.7-sgNC-derived EVs (EVs-sgNC), Raw 264.7-sgPtprc-derived EVs (EVs-sgPtprc), Raw 264.7-sgRab27a-derived EVs (EVs-sgRab27a) (120 μg/mL, 3 μg/mouse) were used with the similar process. For metastatic tumor models, CT26-Vector and CT26-CD45 cells transfected with luciferase plasmid were used to evaluate the extent of tumor metastasis by either (i) using a fixed cell number of 3 × 10^5^ cells per mouse; or (ii) using 10^4^ cells, 10^3^ cells, 10^2^ cells per mouse in a limiting dilution manner, after intracardiac injection of Balb/C mice. Briefly, Balb/C mice were randomly assigned to the control or CD45 groups and anesthetized with trimethylamine (T48402, Sigma-Aldrich) 0.2 mg/g mouse weight. The indicated number of CT26 cells were resuspended in 60 μL PBS and injected intracardially into the circulation of mice. Metastasis onset and growth rate were non-invasively monitored weekly with the In Vivo Imaging System (IS2014N7915, Andor, iKon) during the observation period of the experiment. Hematoxylin and eosin staining (H&E staining) of mouse body tissues was also conducted to evaluate metastasis foci at the time of experiment termination. For the fixed cancer cell number metastasis study, the experimental animals were observed for up to 3 weeks. For the limiting-dilution metastasis study, the formation of lung metastases after intracardiac injection of cancer cells was monitored for up to 10 weeks. For flow cytometric analysis of T cells, single-cell suspensions from tumors were mechanically dissociated using a 70 μm mesh filter. After erythrocyte lysis, fixation and permeabilization, the cell suspensions were blocked with 2% BSA and stained with APC anti-mouse CD3 (BioLegend, 100312) 1:50 dilution, FITC anti-mouse CD8 (BioLegend, 100706) 1:50 dilution, Brilliant Violet 412^TM^ anti-mouse IFN-γ (BioLegend, 505830) 1:50 dilution.

### H&E staining

The paraffin-embedded 4 μm tissue sections were collected, mounted on glass slides, and stained with H&E. The cut sections were deparaffinised in xylene and rehydrated in ethanol. Afterwards, the samples were stained with hematoxylin for 5 min and eosin for 3 min, followed by another immersion in alcohol and xylene. The stained slides were scanned using an automated slide scanner (KF-PRO-020).

### Immunohistochemistry

The paraffin-embedded 4 μm tissue sections were used for immunohistochemistry staining according to the manufacturer’s protocol (KC-RB-035, KangChen Bio-tech). Briefly, samples were immersed in xylene and alcohol several times. Then antigen retrieval was performed using sodium citrate buffer (pH 6.0) in a pressure cooker for 20 min to remove the aldehyde links. Then the endogenic catalase activity was inactivated using 3% hydrogen peroxide solution. The sections were blocked with 3% BSA for 30 min at room temperature before incubation with primary antibodies against CD8 alpha (ab209775, Abcam, 1:100 dilution) or Granzyme B (ab4059, Abcam, 1:100 dilution) overnight at 4 °C and the anti-rabbit IgG/HRP secondary antibody for 20 min at room temperature. The staining was visualized by using a DAB Substrate Kit (ZLI-9017, ZSGB-BIO) according to the manufacturer’s instruction. The stained slides were scanned using the automated slide scanner (KF-PRO-020) and the data were analyzed using HALO Image Analysis Modules.

### FACS sorting CTCs and single-cell CNA sequencing

Each 5 mL peripheral blood from three CRC patients with liver metastasis or multi-organ metastasis was used to isolate interested cells, separately. In addition, 5 mL peripheral blood from a healthy donor was used to set as negative control. After centrifuging at 850 g for 30 min to collect PBMCs using PBMC Isolation Kit, the PBMCs were washed with PBS twice, diluted with 2% FBS PBS and blocked with Human TruStain FcX™ (422301, Biolegend) for 15 min. Then the following antibody were added to the solution and kept on ice in dark for 30 min: (1) PE anti-human CD45 (304008, Biolegend); (2) APC anti-human Lineage Cocktail (CD3/14/16/19/20/56) (348803, Biolegend); (3) FITC anti-human CD326 (FHF326-01-100, 4Abio); (4) Fixable Viability Dye eFluor™ 780 (65-0865-14, eBioscience). Before FACS, ultralow attachment 96-well plates were added 100 μL CTCs culture medium for further in vitro culture and 0.2 mL PCR octuple tubes were added 2 μL PBS with 2% FBS for single-cell CNA sequencing. Three populations of single cells including 65 cells were sorted into the prepared PCR octuple tubes using Overspeed flow cytometer for cell sorting (MoFlo Astrios, Beckman-Coulter): (a) CD45^+^ Lineage-APC^+^ WBCs (Lineage contains CD3/14/16/19/20/56); (b) CD45^+^ Lineage-APC^-^ EpCAM^+^ CTCs; (c) CD45^−^ EpCAM^+^ CTCs. The PCR octuple tubes containing sorted cells were immediately transferred into a box filled with dry ice to keep the cell integrity. The msCNVS™ Medium flux single cell DNA copy number variation library construction and sequencing was completed by SequMed Biotech Inc. Its feature is the use of TN5 transposase assembled with specific barcodes, which can obtain the corresponding data for each sample through these barcode records. After the construction of the library was completed, Qubit2.0 was firstly used for preliminary quantity, and then using Agilent 2100 to detect the length of the insertion fragment of the library, thus ensuring the quality of the library. Once the library is qualified, the different libraries are sequenced on the Illumina Novaseq platform after the demand of the amount of data from the valid concentration and target. The sequencing strategy is PE150 and the sequencing is Paired End dual-end sequencing, of which the length of the reads is 150 bp. Bowtie2 was used for comparing sequencing reads with reference sequences, which uses FM indexes (based on Burrows-Wheler Transform or BWT) to index the genome. Two parameters were used to exclude unqualified sample when performing CNA data: Coefficient of Variation (CV > 0.5) and/or Median Bin Count (MBC < 35). Before calculating the mean copy number and CV values, the following will be removed: ① X/Y chromosome; ② Regions containing CNAs (> 2.5 or < 1); ③ Regions of black list.

### Crystal structure prediction of CD45 homophilic interactions

Rigid protein–protein docking (ZDOCK) was performed with two same human CD45 extracellular regions, domains d1-d4 to study their dimerization. The PDB format of the protein structural domain was downloaded from the Protein Data Bank (PDB) database (http://www.rcsb.org/). The ZDOCK module was run to identify the docking sites and calculate the ZDOCK scores. Comprehensive analysis such as hydrogen bonds was further performed through PDBePISA (https://www.ebi.ac.uk/pdbe/pisa).

### Statistical analysis

The data are presented as means ± SEM. Statistical comparison of experimental groups was determined by unpaired two-tailed Student’s *t*-test or Mann-Whitney *U* test unless otherwise indicated. *P*-value < 0.05 was considered statistically significant. All statistical analyses were conducted by SPSS 22.0 or GraphPad Prism 8.

### Supplementary information

Circulating tumor cells shielded with extracellular vesicle-derived CD45 evade T cell attack to enable metastasis

## Data Availability

The single-cell CNA sequencing data are uploaded onto Sequence Read Archive (SRA) with accession number: PRJNA1082526. Other original data were deposited in the database RDDB2024506602. All original and uncropped films of Western blots were presented at supplementary Figs. 5–7.
