# Supplementary material for: Circulating tumor cells shielded with extracellular vesicle-derived CD45 evade T cell attack to enable metastasis
Source: Signal Transduct Target Ther. 2024 Apr 5;9:84. doi: 10.1038/s41392-024-01789-1 (PMC10995208; doi:10.1038/s41392-024-01789-1)
Supplement: Supplementary file 1 — Circulating tumor cells shielded with extracellular vesicle-derived CD45 evade T cell attack to enable metastasis [file 41392_2024_1789_MOESM1_ESM.docx]

Supplementary Materials for

**Circulating tumor cells shielded with extracellular vesicle-derived CD45 evade T cell attack to enable metastasis**

Chuan Yang^1^, Xueping Wang^1^, Kenneth K.W. To^2^, Caimei Cui^3^, Min Luo^1^, Shaocong Wu^1^, Lamei Huang^1^, Kai Fu^1^, Can Pan^1^, Zeyu Liu^1^, Teng Fan^1^, Caibo Yang^1^, Fang Wang^1*^, Liwu Fu^1*^

Correspondence to: [wangf@sysucc.org.cn](mailto:wangf@sysucc.org.cn) or [fulw@mail.sysu.edu.cn](mailto:fulw@mail.sysu.edu.cn).

**This PDF file includes:**

Supplementary Figures. S1 to S7

Supplementary Tables S1 to S5

**
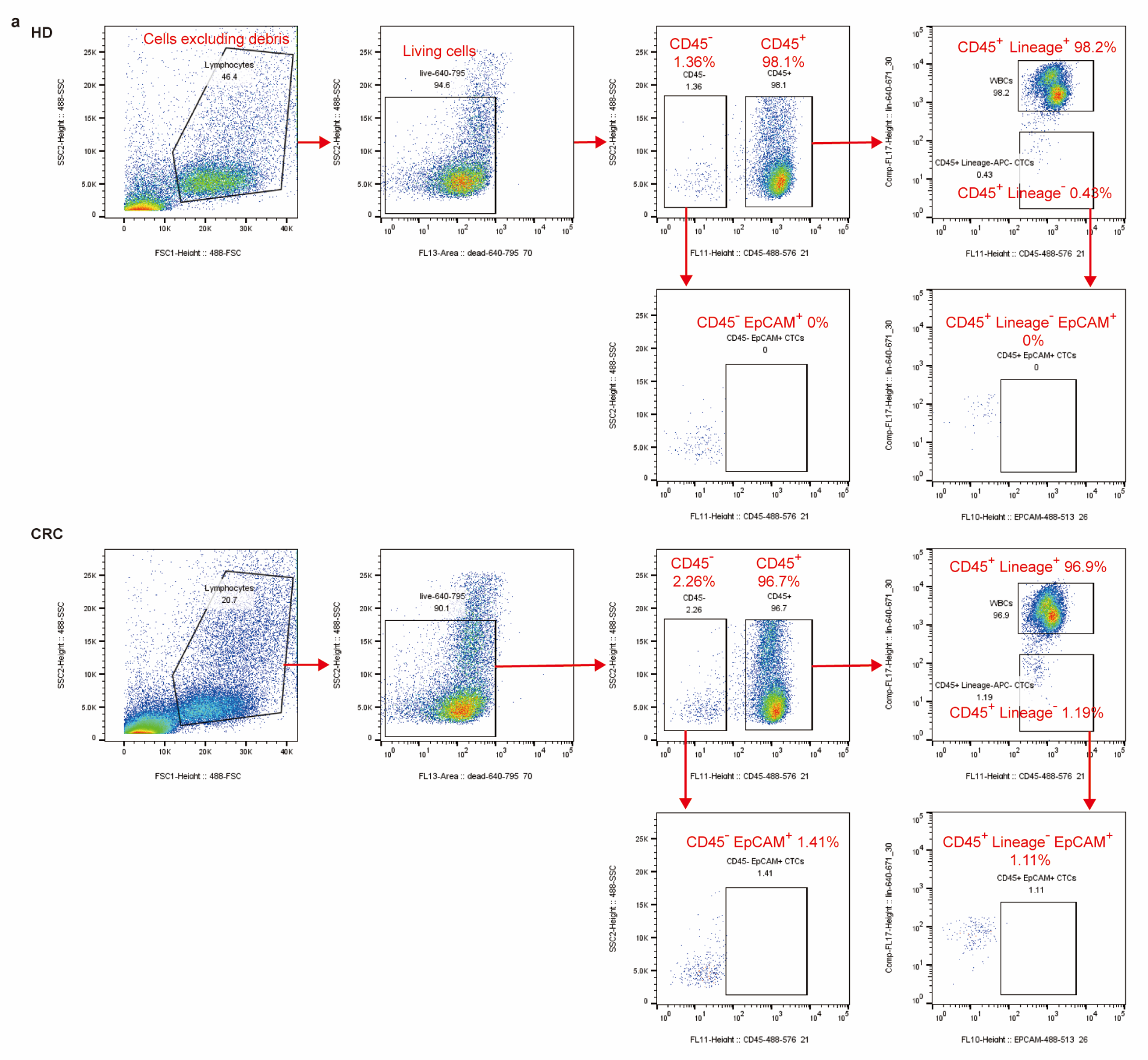
**

**Figure. S1.**

**The identification of CD45^+^ CTCs.**

**a)** Single-cell sorting strategy of (a) CD45^+^ Lineage-APC^+^ WBCs (Lineage contains CD3/14/16/19/20/56); (b) CD45^+^ Lineage-APC^-^ EpCAM^+^ CTCs; (c) CD45^-^ EpCAM^+^ CTCs.


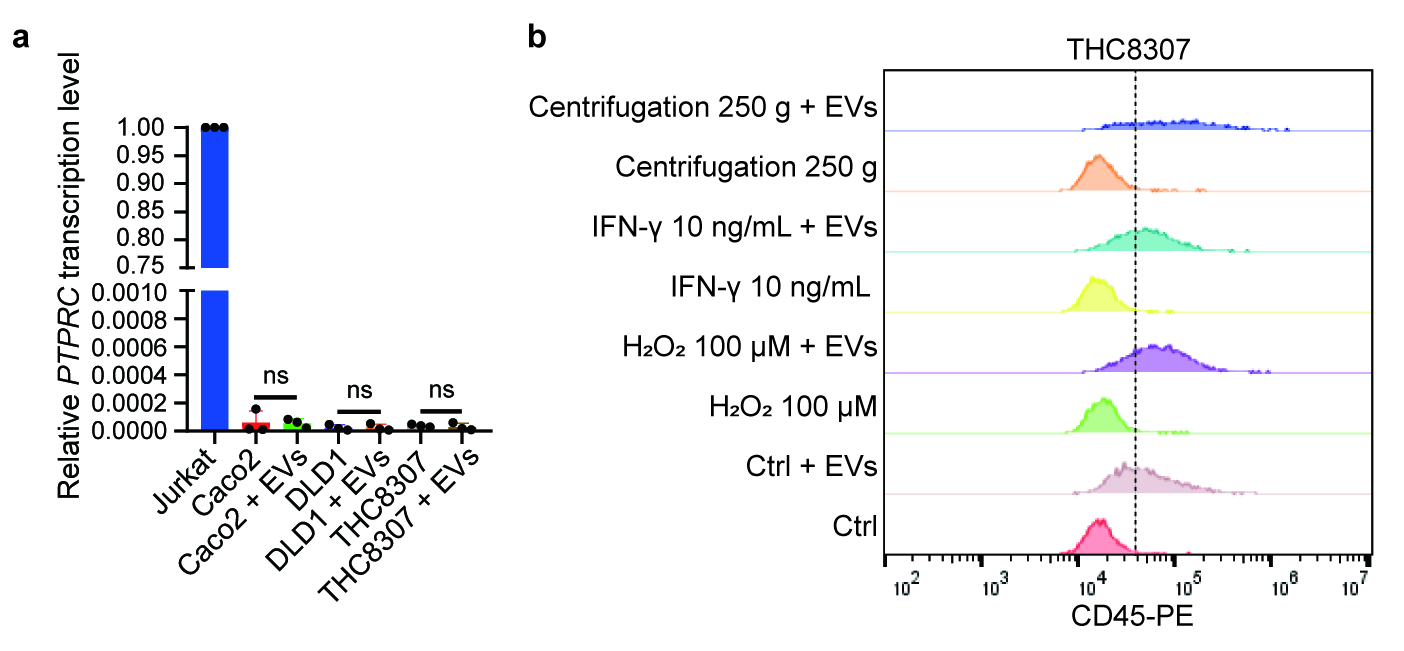


**Figure. S2.**

**EVs can transfer to tumor cells.**

**a)** qRT-PCR analysis of *PTPRC* transcriptional expression. For groups adding EVs, the tumor cells were incubated with Jurkat-derived EVs (4 μg/mL) for 16 h. **b)** Flow cytometry analysis of CD45 uptake using THC8307 cells. Jurkat-derived EVs (20 μg/mL) were used to incubated tumor cells for 12 h. Centrifugation 250 g was simultaneously performed with incubating EVs within 12 h at room temperature.


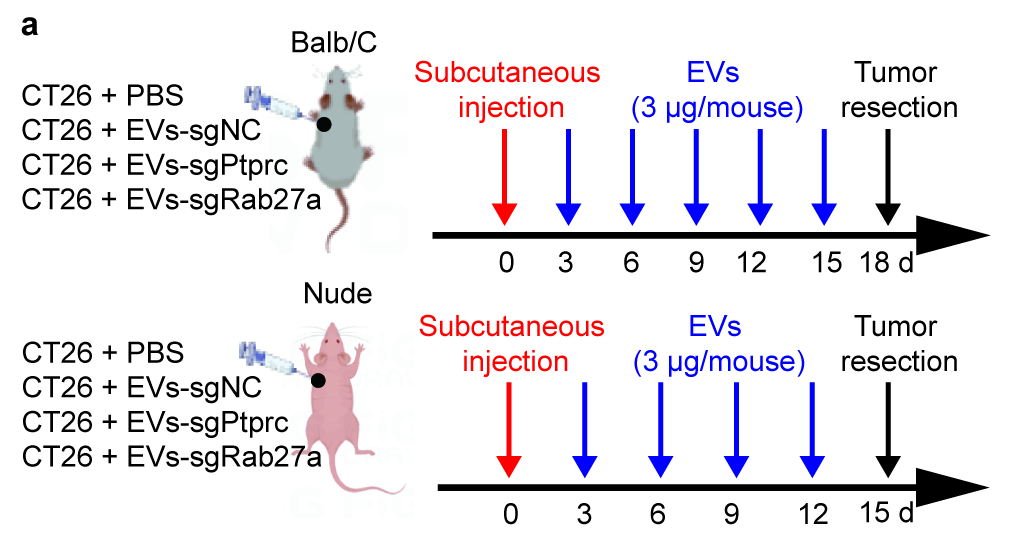


**Figure. S3.**

**Tumor xenografts with EVs injection.**

**a)** Schematic diagram showing the treatment procedure of the subcutaneous tumor model in immunocompetent BALB/c mice or immunodeficient nude mice.


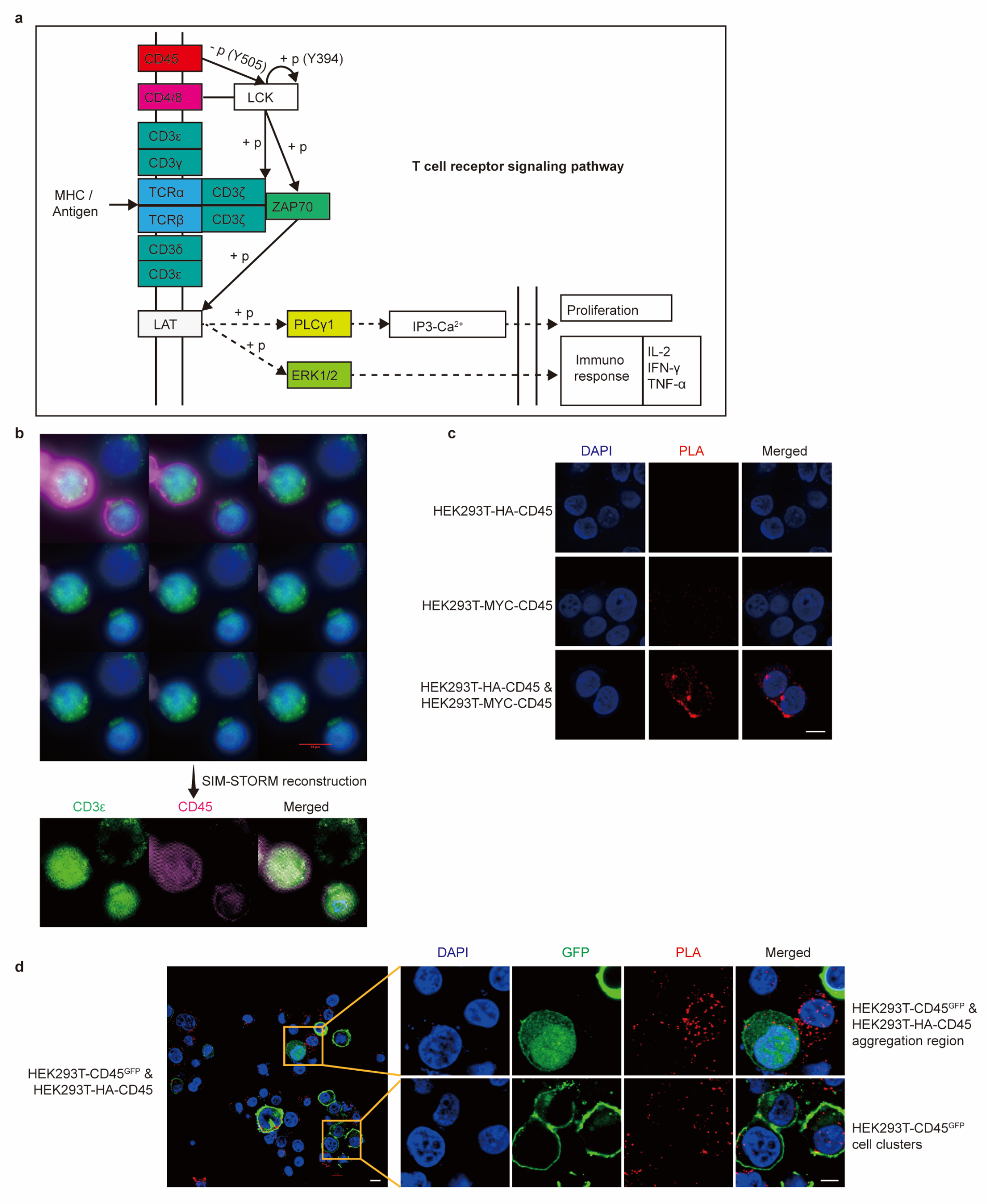


**Figure. S4.**

**Intercellular CD45 homophilic interactions.**

**a)** Diagram of T cell receptor signaling pathway according to information from [*http://www.kegg.jp*](http://www.kegg.jp). + p (phosphorylation), - p (dephosphorylation). **b)** Reconstructed immunofluorescence images captured by N-SIM/N-STORM super-resolution microscope. **c)** Immunofluorescence images showing the intercellular CD45-CD45 homophilic interactions detected by proximity ligation assay. Scale bar = 10 μm. **d)** PLA assay showing the homophilic CD45-CD45 interactions between HEK293T-CD45^GFP^ and HEK293T-HA-CD45. Scale bar = 10 μm.


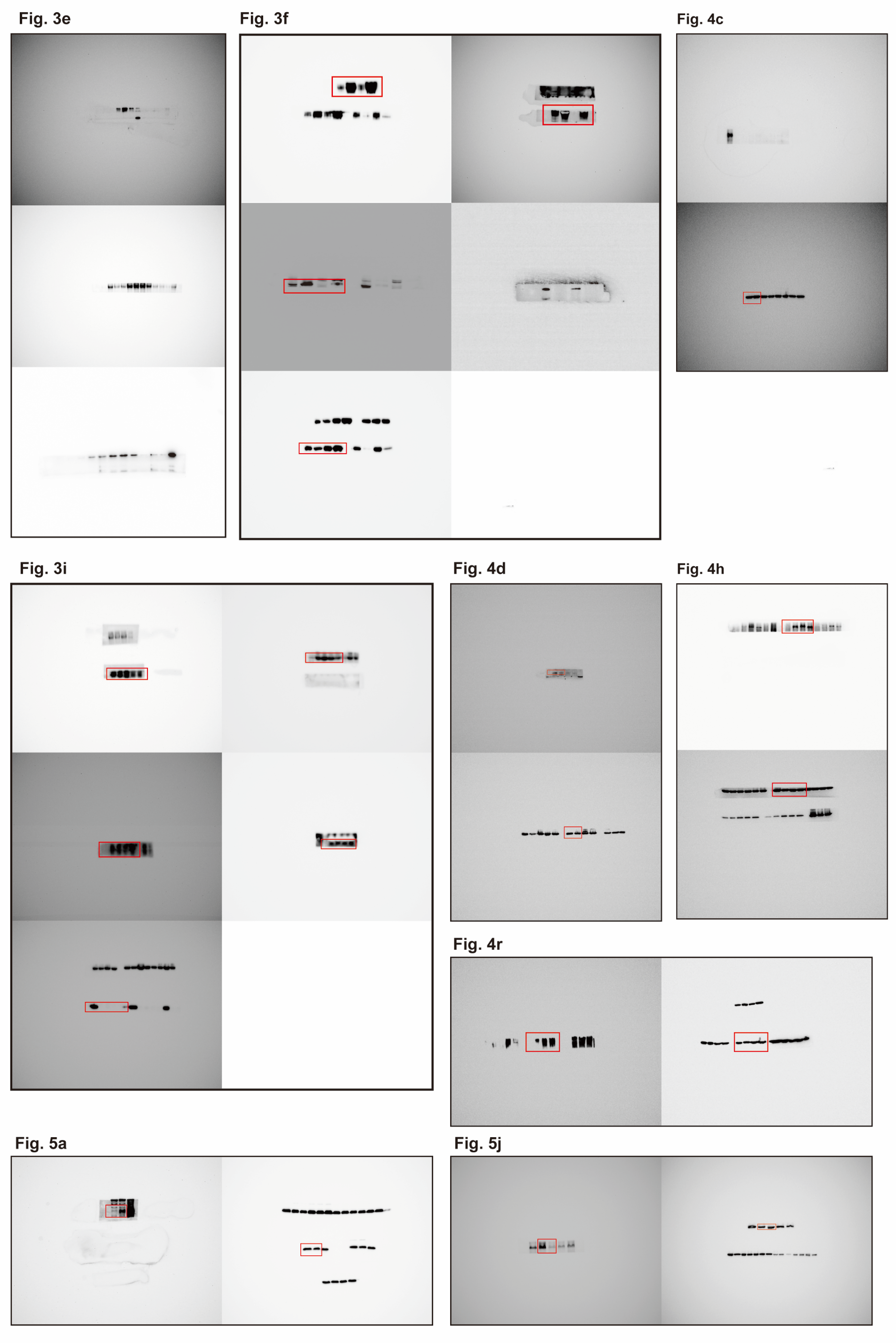


**Figure. S5.**

**The original and uncropped films of Western blots of figure 3 to 5.**


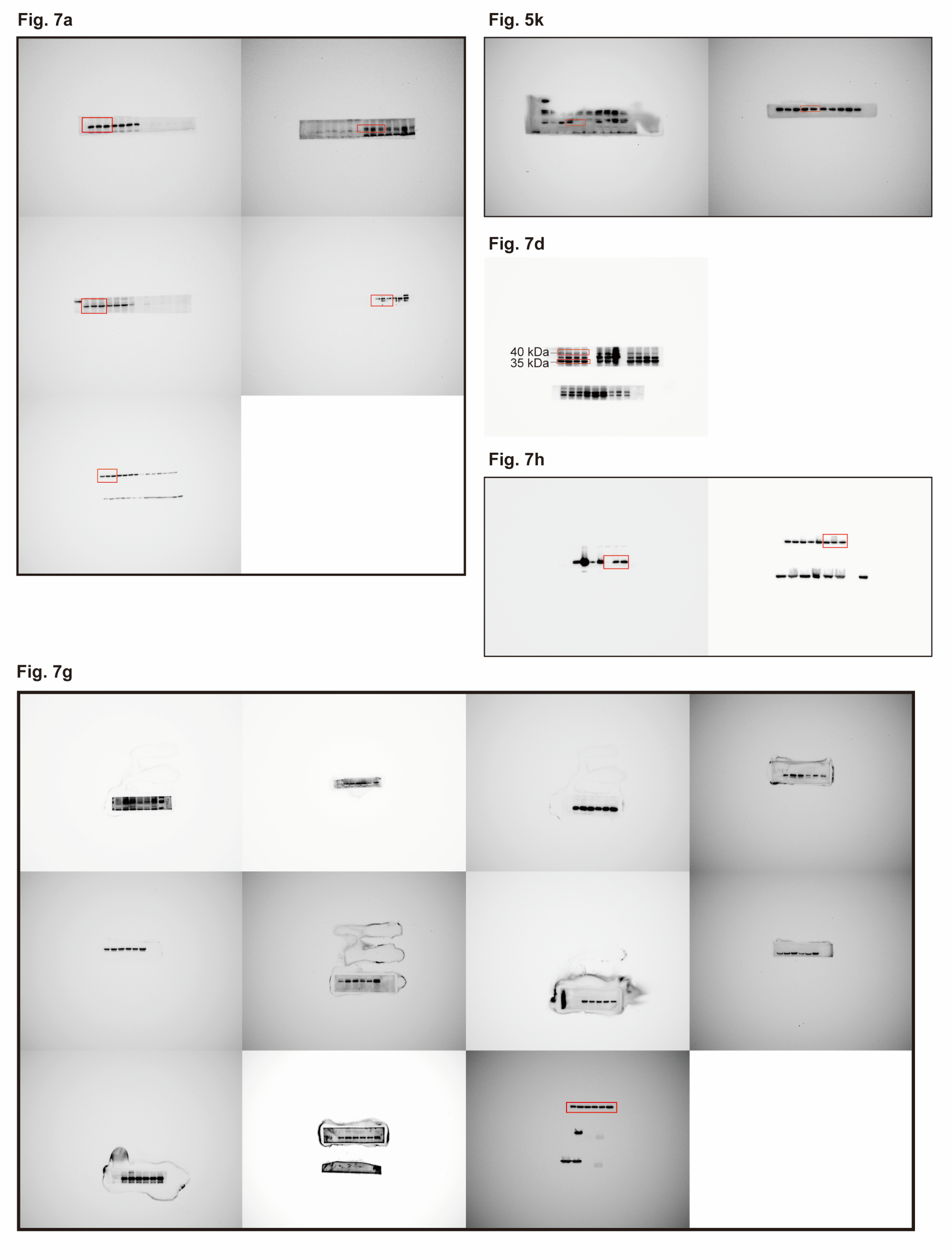


**Figure. S6.**

**The original and uncropped films of Western blots of figure 5 to 7.**


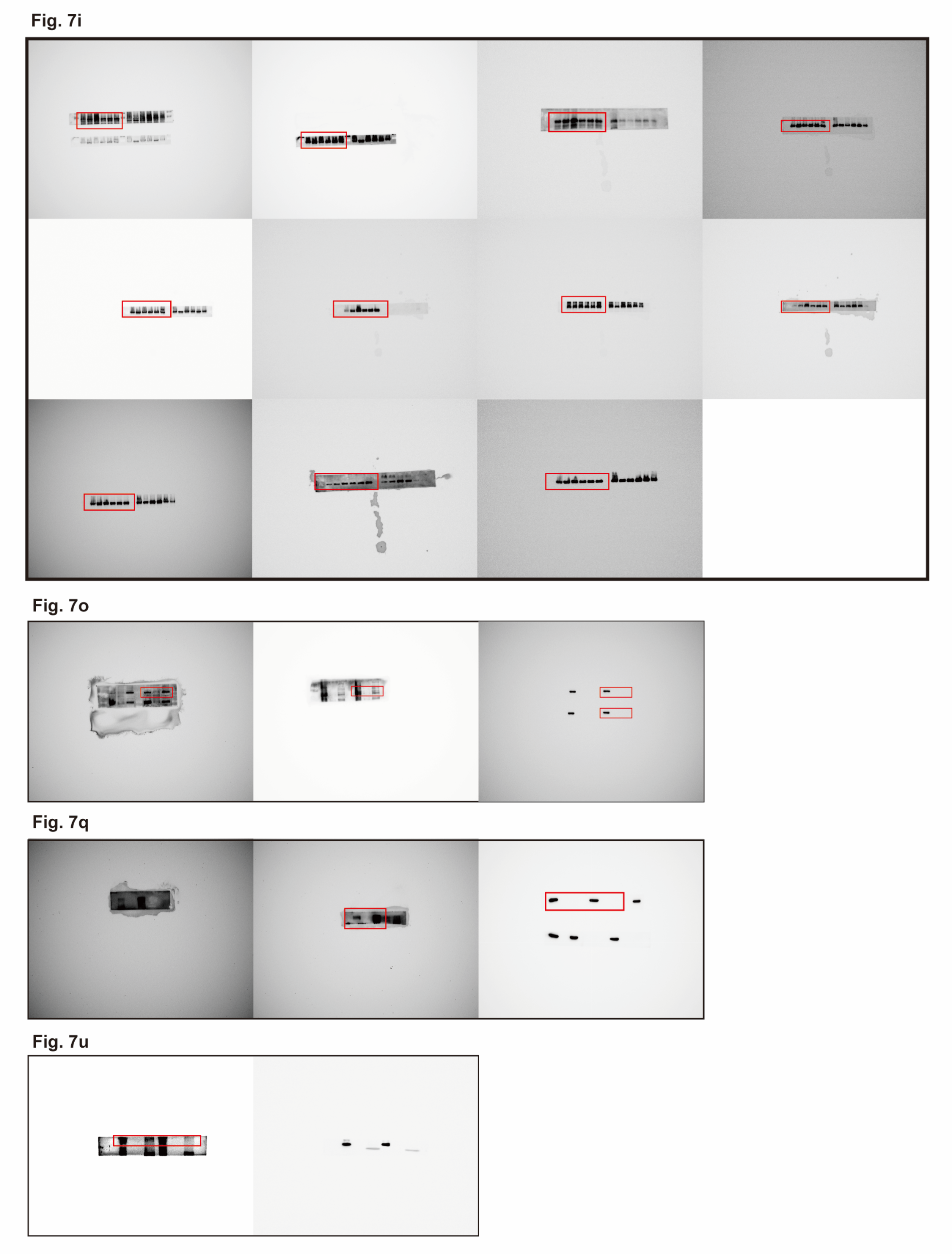


**Figure. S7.**

**The original and uncropped films of Western blots of figure 7.**

**Table S1.** **Correlation between metastasis and clinical characteristics in 144 CRC patients**

| **Variable** | **NO.** | **Pathological characteristics** | | | | ***χ^2^*** | | ***P* value** |
| --- | --- | --- | --- | --- | --- | --- | --- | --- |
|  |  | **Non-metastasis** | | **Metastasis** | |  |  |  |
| **Age** |  |  | |  | |  | |  |
| ≤ 60 | 69 | 39 (56.5%) | | 30 (43.5%) | | 1.567 | | 0.211 |
| > 60 | 75 | 50 (66.7%) | | 25 (33.3%) | |  |  |  |
| **Gender** |  |  | |  | |  | |  |
| Female | 50 | 33 (66.0%) | | 17 (34.0%) | | 0.571 | | 0.450 |
| Male | 94 | 56 (59.6%) | | 38 (40.4%) | |  |  |  |
| **CD45^+^ CTCs (%)** |  | |  |  |  | |  | |
| = 0 | 43 | 36 (83.7%) | | 7 (16.3%) | |  | |  |
| > 0 and < 20 | 44 | 27 (61.4%) | | 17 (38.6%) | | 5.442 | | **0.020**^*^ |
| ≥ 20 and < 40 | 13 | 7 (53.8%) | | 6 (46.2%) | | 4.998 | | **0.025**^*^ |
| > 40 | 44 | 19 (43.2%) | | 25 (56.8%) | | 15.37 | | **< 0.001**^*^ |
| **CD45^+^ CTC counts (5 mL Blood)** | | | | | |  | |  |
| ≤ 1 | 102 | 73 (71.6%) | | 29 (28.4%) | |  | |  |
| = 2 or 3 | 19 | 8 (42.1%) | | 11 (57.9%) | | 6.283 | | **0.012**^*^ |
| ≥ 4 | 23 | 8 (34.8%) | | 15 (65.2%) | | 11.130 | | **0.001**^*^ |

* Each *P* value was determined by comparing with the first group.

**Table S2. Correlation between metastasis and clinical characteristics in 50 NSCLC patients using Cellab ThomasⅠCTCs processing workstation capturing CTCs**

| **Variable** | **NO.** | **Pathological characteristics** | | ***χ^2^*** | ***P* value** |
| --- | --- | --- | --- | --- | --- |
|  |  | **Non-metastasis** | **Metastasis** |  |  |
| **Age** |  |  |  |  |  |
| ≤ 60 | 34 | 20 (58.8%) | 14 (41.2%) | 0.455 | 0.500 |
| > 60 | 16 | 11 (68.7%) | 5 (31.3%) |  |  |
| **Gender** |  |  |  |  |  |
| Female | 25 | 18 (72.0%) | 7 (28.0%) | 2.122 | 0.145 |
| Male | 25 | 13 (52.0%) | 12 (48.0%) |  |  |
| **CD45^-^ CTC counts (5 mL Blood)** | | | | | |
| = 0 | 7 | 6 (85.7%) | 1 (14.3%) | 0.949 | 0.330 |
| ≥ 1 | 43 | 25 (58.1%) | 18 (41.9%) |  |  |
| **CD45^+^ CTC counts (5 mL Blood)** | | | |  |  |
| = 0 | 25 | 21 (84.0%) | 4 (16.0%) | 10.27 | **0.001** |
| ≥ 1 | 25 | 10 (40.0%) | 15 (60.0%) |  |  |

**Table S3. Correlation between metastasis and clinical characteristics in 62 NSCLC patients using NextCTC capturing CTCs**

| **Variable** | **NO.** | **Pathological characteristics** | | ***χ^2^*** | ***P* value** |
| --- | --- | --- | --- | --- | --- |
|  |  | **Non-metastasis** | **Metastasis** |  |  |
| **Age** |  |  |  |  |  |
| ≤ 60 | 33 | 8 (24.2%) | 25 (75.8%) | 0.090 | 0.764 |
| > 60 | 29 | 8 (27.6%) | 21 (72.4%) |  |  |
| **Gender** |  |  |  |  |  |
| Female | 23 | 7 (30.4%) | 16 (69.6%) | 0.409 | 0.522 |
| Male | 39 | 9 (23.1%) | 30 (76.9%) |  |  |
| **CD45^-^ CTC counts (5 mL Blood)** | | | | | |
| = 0 | 48 | 14 (29.2%) | 34 (70.8%) | 0.597 | 0.440 |
| ≥ 1 | 14 | 2 (14.3%) | 12 (85.7%) |  |  |
| **CD45^+^ CTC counts (5 mL Blood)** | | | |  |  |
| = 0 | 16 | 10 (62.5%) | 6 (37.5%) | 15.160 | **< 0.001** |
| ≥ 1 | 46 | 6 (13.0%) | 40 (87.0%) |  |  |

**Table S4. Correlation between metastasis and clinical characteristics in 64 HCC patients using NextCTC capturing CTCs**

| **Variable** | **NO.** | **Pathological characteristics** | | ***χ^2^*** | ***P* value** |
| --- | --- | --- | --- | --- | --- |
|  |  | **Non-metastasis** | **Metastasis** |  |  |
| **Age** |  |  |  |  |  |
| ≤ 60 | 51 | 36 (70.6%) | 15 (29.4%) | 0.060 | 0.807 |
| > 60 | 13 | 9 (69.2%) | 4 (30.8%) |  |  |
| **Gender** |  |  |  |  |  |
| Female | 8 | 8 (100%) | 0 (0%) | 2.406 | 0.121 |
| Male | 56 | 37 (66.1%) | 19 (33.9%) |  |  |
| **CD45^-^ CTC counts (5 mL Blood)** | | | | | |
| = 0 | 48 | 37 (77.1%) | 11 (22.9%) | 4.217 | **0.040** |
| ≥ 1 | 16 | 8 (50.0%) | 8 (50.0%) |  |  |
| **CD45^+^ CTC counts (5 mL Blood)** | | | |  |  |
| = 0 | 20 | 18 (90.0%) | 2 (10.0%) | 4.117 | **0.043** |
| ≥ 1 | 44 | 27 (61.4%) | 17 (38.6%) |  |  |

**Table S5. Hydrogen bonds from crystal structure prediction of human CD45 extracellular region, domains d1-d4**

| **##** | **CD45 # 1** | **Dist [Å]** | **CD45 # 2** |
| --- | --- | --- | --- |
| **1** | A:LYS 430[ N ] | 2.72 | B:LYS 430[ O ] |
| **2** | A:CYS 432[ N ] | 3.06 | B:CYS 432[ O ] |
| **3** | A:ASN 524[ ND2 ] | 2.89 | B:THR 333[ O ] |
| **4** | A:ASN 524[ ND2 ] | 3.56 | B:ILE 336[ O ] |
| **5** | A:THR 525[ OG1 ] | 3.64 | B:THR 333[ OG1 ] |
| **6** | A:THR 428[ O ] | 2.99 | B:LYS 430[ N ] |
| **7** | A:GLU 429[ OE2 ] | 3.60 | B:LYS 430[ NZ ] |
| **8** | A:LYS 430[ O ] | 2.69 | B:CYS 432[ N ] |
| **9** | A:CYS 432[ O ] | 3.73 | B:ASN 434[ N ] |
| **10** | A:ASN 446[ OD1 ] | 3.79 | B:GLN 466[ N ] |
